# Vaginal Laser Therapy for Genitourinary Syndrome of Menopause in Breast Cancer Survivors: A Modality-Specific Systematic Review of Efficacy, Durability, and Safety

**DOI:** 10.3390/jcm15197473

**Published:** 2026-09-25

**Authors:** Angel Yordanov, Eva Tsoneva, Vasilena Dimitrova Dimitrova, Stoyan Kostov, Zlatko Kirovakov, Ihsan Hasan

**Affiliations:** 1 Department of Gynaecological Oncology, Medical University Pleven, 5800 Pleven, Bulgaria; angel.jordanov@gmail.com (A.Y.);; 2 Gynecology Clinic, Military Medical Academy, 1606 Sofia, Bulgaria; 3 Research Institute, Medical University Pleven, 5800 Pleven, Bulgaria; drstoqn.kostov@gmail.com; 4 Faculty for Public Health and Healthcare, Burgas State University Prof. Dr. Assen Zlatarov, 8010 Burgas, Bulgaria; kirovakov@yahoo.com; 5 Department of Gynaecology and Pelvic Surgery, Hospital (MHAT), Vita 2, 1407 Sofia, Bulgaria; ihsan_hasanov@abv.bg

**Keywords:** breast cancer survivors, genitourinary syndrome of menopause, vaginal laser, CO_2_ laser, Er:YAG laser, systematic review

## Abstract

**Background**: Genitourinary syndrome of menopause (GSM) is common among breast cancer survivors, particularly during endocrine therapy, and vaginal laser therapy has been proposed as a nonhormonal option; CO_2_ and Er:YAG devices differ technically and their effects should not be assumed equivalent. **Objective**: To assess the efficacy, durability, and safety of vaginal laser therapy for GSM in breast cancer survivors, treating CO_2_ and Er:YAG separately. **Methods**: Five databases and trial registries were searched from inception to 22 August 2026. Risk of bias was assessed with RoB 2, ROBINS-I, and the JBI cohort checklist; certainty with GRADE; and findings were synthesized narratively because of heterogeneity. **Results**: Thirty-five evidence reports represented 28 study families (19 CO_2_, six Er:YAG alone, one Er:YAG plus Nd:YAG versus Er:YAG, two other systems). Only two CO_2_ trials used sham controls, and neither showed a reproducible laser-specific benefit for dyspareunia or overall sexual function; one favored CO_2_ for daily dryness and selected objective measures but not intercourse dryness or dyspareunia. Active-comparator trials showed no consistent laser advantage, and uncontrolled cohorts reported short-term gains that attenuated with follow-up. No serious treatment-related adverse event was reported in the included studies; however, the evidence was insufficient to assess uncommon or delayed harms. Certainty was low to very low for sham-controlled CO_2_ effects and very low for Er:YAG versus hyaluronic acid; no study compared the modalities directly. **Conclusions**: Available evidence does not establish a consistent laser-specific benefit on the main patient-reported outcomes. CO_2_ has the larger evidence base, but this does not demonstrate greater efficacy; Er:YAG data rely mainly on active-comparator or uncontrolled studies. No direct CO_2_-versus-Er:YAG comparison is available, and neither modality superiority nor equivalence can be inferred. Durability and safety beyond the short term remain uncertain.

## 1. Introduction

Genitourinary syndrome of menopause (GSM) is an umbrella term for the genital, sexual, and urinary symptoms that follow estrogen loss, among them vaginal dryness, burning, irritation, dyspareunia, dysuria, and recurrent urinary tract infections. Unlike hot flashes and other transient menopausal complaints, GSM tends to persist and often worsens without effective treatment [1,2].

The issue is especially pressing for breast cancer survivors, in whom GSM symptoms can undermine sexual function, intimate relationships, quality of life, and even adherence to long-term cancer therapy [3,4].

How severe GSM becomes in these women depends on an interplay of age, menopausal status, and the anticancer treatment itself (Figure 1). Older survivors often already carry some degree of postmenopausal estrogen deficiency, whereas younger women can be pushed into abrupt treatment-induced menopause by chemotherapy or ovarian suppression. Aromatase inhibitors compound this by further reducing peripheral estrogen synthesis, which can worsen dryness, epithelial thinning, loss of elasticity, elevated vaginal pH, and dyspareunia. Tamoxifen’s tissue-specific effects are more heterogeneous, but genitourinary symptoms can still occur during treatment. GSM in breast cancer survivors therefore reflects both ordinary reproductive ageing and treatment-related estrogen deprivation, whether gradual or abrupt [3,4].

First-line management usually consists of nonhormonal moisturizers and lubricants, but relief from these is often partial and short-lived. Local estrogen can help, yet prescribing it is far from straightforward in women with hormone-sensitive breast cancer, particularly those receiving aromatase inhibitors [2]. This gap in effective, low-risk options has driven interest in energy-based devices, chiefly fractional carbon dioxide (CO_2_) and erbium:YAG vaginal lasers.

Two device types dominate the evidence in this area: fractional or microablative CO_2_ laser and Er:YAG laser, the latter typically delivered through non-ablative or subablative SMOOTH protocols [5,6]. They interact with tissue differently, use different delivery parameters and treatment schedules, and for that reason their results should be interpreted separately rather than treated as interchangeable.

Early uncontrolled series reported improvement in vaginal symptoms and tissue measures after laser treatment, but the comparative picture has been less consistent. One randomized trial comparing Er:YAG with hyaluronic acid reported improvement in both arms but did not detect a between-group difference; its small sample leaves clinically important differences uncertain [5]. Sham-controlled trials, too, have failed to show a clear laser advantage for several core patient-reported outcomes [6,7]. More recent work has broadened the evidence base with new comparators, treatment schedules, and longer follow-up, yet how large any benefit is, how long it lasts, and whether it is truly laser-specific remain open questions [8,9].

We therefore set out to evaluate the efficacy, durability, and safety of vaginal laser therapy for GSM in breast cancer survivors, treating CO_2_ and Er:YAG as distinct interventions throughout. Comparative and longitudinal evidence is synthesized by laser modality, comparator type, endocrine therapy status, and follow-up duration.

## 2. Materials and Methods

### 2.1. Review Design and Reporting Framework

This review follows the Preferred Reporting Items for Systematic Reviews and Meta-Analyses (PRISMA) 2020 statement [10], and a completed PRISMA 2020 checklist is provided as a Supplementary File. The review protocol was finalized on 21 August 2026, immediately before title screening commenced later that day. The PROSPERO record was created on 22 August 2026 and registered on 23 August 2026 (CRD420261485605). Before screening, the protocol specified the eligibility criteria, primary and secondary outcomes, separate analysis of CO_2_ and Er:YAG modalities, the three evidence streams (comparative efficacy, durability, and safety), the risk-of-bias and certainty-assessment methods, and conditional meta-analysis only when studies were clinically and methodologically compatible. No substantive protocol deviations occurred between the start of screening and registration or after registration. Subsequent amendments were limited to refinement of the review title and terminology, correction of study-design classifications, and alignment of reporting across the manuscript and Supplementary Materials; these changes did not alter eligibility, outcomes, modality grouping, or synthesis rules. The decision not to pool outcomes was therefore an application of the prespecified compatibility criterion rather than a post hoc change in the synthesis plan.

### 2.2. Eligibility Criteria

Eligible studies enrolled adult women with a past or current breast cancer diagnosis who had symptoms or clinical findings consistent with GSM, vulvovaginal atrophy, or urogenital atrophy arising from natural or treatment-induced hypoestrogenism. Where a study enrolled a mixed oncological or postmenopausal population, it was included only if breast cancer survivor data could be extracted separately.

Eligible interventions covered intravaginal or vulvovaginal laser therapy of any type or protocol, including CO_2_, Er:YAG, combined Er:YAG–Nd:YAG, diode, and other solid-state vaginal laser systems. For the comparative-efficacy analysis, eligible comparators included sham or placebo procedures, no treatment, usual care, moisturizers or lubricants, hyaluronic acid, promestriene or other topical agents, pelvic-floor interventions, radiofrequency, or other active treatments.

Randomized controlled trials and prospective comparative studies qualified for the comparative-efficacy analysis, while randomized and non-randomized comparative studies plus longitudinal single-arm cohorts qualified for the durability and safety analyses. We excluded systematic or narrative reviews, meta-analyses, editorials, commentaries, case reports, technical reports, animal or in vitro studies, and any study whose breast cancer data could not be extracted separately. Trial protocols and registry records were retained for identifying and linking studies but counted as efficacy evidence only once results became available.

Case reports were excluded because isolated observations without a defined cohort denominator or systematic follow-up cannot provide reliable estimates of comparative efficacy, durability, or safety. Technical reports without extractable clinical outcomes were excluded for the same reason. Small uncontrolled cohorts remained eligible when they enrolled a defined breast-cancer-survivor population, applied a longitudinal assessment, and reported extractable clinical outcomes.

The primary outcomes were patient-reported vaginal dryness and dyspareunia. Secondary outcomes spanned sexual function, vaginal health and maturation indices, vaginal pH, urinary symptoms, health-related quality of life, treatment satisfaction, and adverse events. Durability was operationally defined for this review as persistence of a reported outcome at ≥6 months from treatment initiation. Follow-up at ≥12 months was categorized as extended follow-up. These thresholds were used solely to organize the evidence and do not imply established long-term treatment efficacy or safety.

### 2.3. Information Sources and Search Strategy

We searched PubMed/MEDLINE, Scopus, and the Cochrane Central Register of Controlled Trials (CENTRAL), and looked separately for ongoing, completed, and unpublished studies in ClinicalTrials.gov and the World Health Organization International Clinical Trials Registry Platform (WHO ICTRP). All five sources were searched from inception, with the final searches run on 21–22 August 2026.

Search strings combined controlled vocabulary, where each database offered it, with free-text terms for breast cancer and laser therapy. GSM-specific terms were deliberately not required, since a report can identify the right population and intervention without using standardized GSM language in its title, abstract, or registry entry. No restrictions were placed on language, publication year, document type, or subject area, and syntax was adapted to the indexing and interface of each source. Full, reproducible search strategies, together with the date and yield of every search, are provided in Supplementary Table S1.

### 2.4. Study Selection

After removing duplicates, two reviewers independently screened titles and abstracts against the eligibility criteria and then independently assessed the full texts of potentially eligible reports. Disagreements at either stage were settled by discussion or, when necessary, referred to a third reviewer, and reasons for full-text exclusion were recorded. Multiple publications, conference abstracts, and registry records tied to the same underlying study were linked and treated as a single study family. The selection process is summarized in a PRISMA 2020 flow diagram [10].

### 2.5. Data Extraction

Two reviewers independently extracted data using a piloted form covering study identifiers, country and setting, design, recruitment period, sample size, participant age and menopausal status, breast cancer characteristics, endocrine and other anticancer therapy, GSM diagnostic criteria, laser modality and technical parameters, number and timing of sessions, comparator details, outcome definitions and instruments, assessment time points, numerical results, follow-up duration and attrition, adverse events, and funding or conflicts of interest. Laser modality was classified as CO_2_, Er:YAG, combined Er:YAG–Nd:YAG, diode, or another solid-state system. For CO_2_ studies specifically, fractional or microablative status and other technical descriptors were recorded exactly as the original authors reported them, without treating author-supplied terminology as defining a separate modality. Where available, wavelength, ablative profile, energy settings, number and interval of sessions, and vaginal versus vulvar application were also extracted. Disagreements were resolved by consensus or a third reviewer, and reports describing the same population were grouped to avoid double counting, drawing on companion publications and registry entries to fill gaps.

### 2.6. Risk-of-Bias Assessment

Two reviewers assessed risk of bias independently, using RoB 2 for randomized trials and ROBINS-I for non-randomized studies estimating comparative intervention effects [11,12]. ROBINS-I was reserved for such studies because it evaluates bias in causal estimates that compare interventions. The JBI Critical Appraisal Checklist for Cohort Studies was used for non-comparative longitudinal cohorts and observational subgroup evidence contributing mainly to the durability and safety streams [13]. Disagreements again went to consensus or a third reviewer, and the resulting judgments were taken into account when interpreting findings and assessing certainty.

### 2.7. Data Synthesis and Statistical Analysis

Comparative efficacy, durability, and safety were synthesized as separate strands, with randomized and non-randomized evidence kept apart and uncontrolled cohorts never pooled with randomized comparisons. Sham-controlled comparisons were likewise distinguished from active-comparator and no-treatment comparisons.

Evidence was further organized by laser modality: CO_2_ and Er:YAG studies were synthesized on separate tracks, and combined Er:YAG–Nd:YAG, diode, and other solid-state systems were described on their own. Because no robust head-to-head trial compared CO_2_ with Er:YAG directly, any apparent cross-modality difference was treated as descriptive only and not as evidence that one modality outperforms the other.

For continuous outcomes measured on the same scale, the prespecified effect measure was the mean difference with 95% confidence intervals; standardized mean differences were planned for conceptually similar outcomes measured on different scales, and risk ratios with 95% confidence intervals for dichotomous outcomes. Change scores and post-intervention values were combined only where methodologically appropriate.

When a valid between-group effect estimate and its variance were neither reported nor calculable, the reported arm-level results, model coefficients, or medians were retained rather than converted into a non-equivalent effect measure. Thus, mean differences, standardized mean differences, and risk ratios were implemented only where the available data supported them.

Meta-analysis was planned only where at least two studies were sufficiently similar in population, intervention, comparator, outcome definition, and follow-up, using a random-effects model given the clinical and methodological heterogeneity expected from the outset. Statistical heterogeneity would have been evaluated with the I^2^ statistic and between-study variance alongside clinical judgment; where quantitative pooling was inappropriate, findings were instead synthesized narratively and presented in structured tables.

Where the data allowed, prespecified subgroups included laser modality, comparator type, endocrine therapy status, and follow-up duration, and sensitivity analyses were planned by study design, risk of bias, outcome measurement approach, and possible population overlap. Selective outcome reporting was examined by comparing publications against available protocols or registry records, and funnel plots and statistical tests for small-study effects were reserved for outcomes with at least 10 sufficiently comparable studies.

Because several eligible results were available to the review team only as detailed abstracts or registry results, we additionally performed a post hoc qualitative sensitivity analysis restricted to outcome evidence available in full-text, peer-reviewed reports. This analysis assessed whether exclusion of abstract-only and registry-results-only findings altered the direction or certainty of the principal conclusions.

### 2.8. Certainty of Evidence

Certainty of evidence was assessed with GRADE for each intervention-comparator-outcome question, weighing risk of bias, inconsistency, indirectness, imprecision, and publication bias [14]. Sham-controlled CO_2_ comparisons were used to assess laser-specific efficacy because they isolate the procedural effect from contextual and placebo effects. GRADE was not restricted to sham-controlled evidence: the randomized Er:YAG-versus-hyaluronic-acid comparison was rated separately as a comparative-effectiveness question against an active nonhormonal treatment. Its certainty applies only to the relative effect of Er:YAG versus hyaluronic acid and not to a laser-specific effect versus sham or no treatment. Other active-comparator studies evaluated different treatments or laser protocols and were synthesized separately rather than merged with either of these GRADE bodies. When a comparison was informed by a single study, inconsistency was recorded as not assessable and was not itself a reason for downgrading. Imprecision was judged from the sample size, the availability and width of confidence intervals, and whether clinically important benefit or harm could be excluded. When only arm-level medians, interquartile ranges, and *p*-values were reported, the absence of a between-group effect estimate with a 95% confidence interval was considered together with sample size rather than as a separate GRADE domain.

### 2.9. Use of Generative Artificial Intelligence

During the preparation and revision of this manuscript, the authors used ChatGPT (GPT-5 series; OpenAI, San Francisco, CA, USA; accessed August–September 2026) to support English-language editing, improve clarity and consistency, and assist with the organization and formatting of selected text and Supplementary Tables. All study-selection decisions, extracted data, risk-of-bias assessments, GRADE judgments, and scientific interpretations were critically verified and approved by the authors.

## 3. Results

### 3.1. Study Selection

Database and registry searches identified 557 records: 85 from PubMed/MEDLINE, 137 from Scopus, 224 from CENTRAL, 17 from ClinicalTrials.gov, and 94 from the WHO International Clinical Trials Registry Platform. Removing 163 duplicates left 394 unique records for title and abstract screening; two reviewers screened all records independently. After their independent judgments were compared, agreement was reached for every record and no case required third-reviewer adjudication. Sixty-three records went forward to full-text retrieval, and 331 were excluded at this stage.

One of the 63 reports sought could not be retrieved, leaving 62 assessed for eligibility; 27 were excluded, 11 registry or protocol records with no eligible results, two reports from mixed populations whose breast cancer data could not be separated out, and 14 superseded conference abstracts or registry companion records. This left 49 records linked to the included study families, of which 14 were linkage-only protocols, registry entries, or superseded conference reports contributing no independent results. The remaining 35 evidence reports, 34 published articles plus one registry-results-only report, represented, after linking companion publications, 28 distinct study families: 27 backed by published articles and one additional registry-results-only study (Figure 2).

### 3.2. Study Characteristics

Table 1 summarizes the 28 included study families: 19 evaluated CO_2_ laser, six evaluated Er:YAG alone, one compared combined Er:YAG–Nd:YAG with Er:YAG alone, one evaluated a dual-wavelength diode laser, and one used another solid-state system. Five families were randomized, three CO_2_ and two Er:YAG, but only the two CO_2_ trials had a sham comparator, and none compared CO_2_ with Er:YAG directly. Multiple reports arising from the same cohort or trial were consolidated to prevent double counting; the registry-results-only study is presented separately from the 27 families with published articles. Table 1 is restricted to the core study characteristics; device-specific and protocol descriptors are provided in Supplementary Table S11.

#### Endocrine-Therapy Representation and Subgroup Evidence

Endocrine-therapy exposure was common but seldom analyzed as an effect modifier. LIGHT enrolled only aromatase-inhibitor users, while LARF/EPMLARF included women on tamoxifen or aromatase inhibitors; neither trial compared response across endocrine regimens [6,8,15,16]. Veron reported 36/46 participants on endocrine therapy (tamoxifen *n* = 6, aromatase inhibitor *n* = 24, and aromatase inhibitor plus LHRH agonist *n* = 6), but no regimen-specific outcomes [24]. Salvatore found improvement in both current- and previous-therapy groups without an endpoint difference; Lami reported improvement irrespective of aromatase inhibitor, tamoxifen, or no therapy but provided no arm-level estimates in the available abstract; and Gittens/Mullen reported within-group improvement in only eight endocrine-treated survivors [25,26,39,42]. Chung included 11 tamoxifen users, one aromatase-inhibitor user, and 12 women without endocrine therapy but did not provide a reliable subgroup comparison [36]. No study was randomized or adequately powered to determine whether response differs among aromatase inhibitors, tamoxifen, ovarian suppression, and no current endocrine therapy (Supplementary Table S10).

### 3.3. Vaginal Dryness and Dyspareunia

Across reported samples, 17 study families (approximately 596 participants) contributed vaginal-dryness data and 22 study families (approximately 1007 participants) contributed dyspareunia or closely related sexual-pain data. These totals use the largest relevant breast-cancer-survivor denominator per family; outcome-specific denominators were sometimes smaller, and possible cohort overlap means that the totals should not be interpreted as counts of unique individuals.

Among the randomized CO_2_ trials, LIGHT found no difference from sham in dyspareunia change at 6 months (mean difference −0.3, 95% CI −1.9 to 1.3; *p* = 0.73) [6]. Jaber found much the same for dyspareunia (GEE coefficient 0.12 ± 0.43; *p* = 0.777) and intercourse dryness (−0.03 ± 0.40; *p* = 0.944) one month after three sessions, though daily dryness did favor laser (−1.30 ± 0.55; *p* = 0.017) [7]. In LARF/EPMLARF, moderate/severe dryness and dyspareunia both improved over time, but the group-by-time interaction was no different across CO_2_ laser, radiofrequency, and promestriene through 10 months [8]. In Gold, dryness-bother NRS medians fell from 7 (IQR 3−8) to 3 (0–7) with Er:YAG and from 4 (0–8) to 2 (0–4) with hyaluronic acid; the 3-month comparison did not detect a difference (*p* = 0.411). Dyspareunia-bother medians fell from 9 (8–10) to 7 (1–9) and from 7 (5–10) to 5 (0–8), respectively (*p* = 0.216) [5]. The Fidecicchi pilot, assessed from the detailed abstract available to the review team, reported a larger reduction in superficial dyspareunia with hyperstack treatment than standard VEL (73.5% vs. 58%; *p* < 0.001), though without arm-level variance estimates [18]. No trial compared CO_2_ with Er:YAG directly, so these modality-specific findings do not establish which performs better.

Most uncontrolled CO_2_ cohorts reported striking within-group improvement in the short term, though such estimates cannot separate a genuine treatment effect from contextual factors, regression to the mean, or the natural fluctuation of GSM symptoms. In the Ohio State cohort, moderate/severe dryness fell from 88.1% to 30.5% and moderate/severe dyspareunia from 81.2% to 30.5% four weeks after treatment [22], and similarly large short-term reductions appeared in the Salvatore and LAAVA cohorts [25,28]. Longer-term CO_2_ findings were more mixed: Favier found dryness improvement persisted beyond 24 months but dyspareunia did not change significantly [23], while in the Ohio State cohort the combined Vaginal Assessment Scale stayed broadly stable from 4 weeks to 2 years among the 31 participants with paired data, though dryness and dyspareunia were not reported separately at that final point [20]. For Er:YAG, Gambacciani and Levancini reported early improvement that was no longer statistically different from baseline by 18 months [30,31]. None of these uncontrolled comparisons can establish whether CO_2_ and Er:YAG actually differ.

No meta-analysis was attempted for either dryness or dyspareunia. The two sham-controlled CO_2_ trials differed in treatment schedule, follow-up, and analytic approach, and the Er:YAG evidence relied on active comparators or divergent Er:YAG strategies. The remaining comparative studies used thresholded outcomes or different comparator structures, and most observational studies were uncontrolled and lacked variance estimates for within-participant change. Complete modality-labelled study-level results, and the reasoning behind each non-pooling decision, are provided in Supplementary Table S5. For the two sham-controlled CO_2_ trials specifically, LIGHT did not report a stand-alone vaginal-dryness outcome corresponding to Jaber’s separate daily and intercourse-dryness constructs. Although both trials assessed dyspareunia on a 0–10 VAS, LIGHT reported a between-group difference in change 6 months after five monthly sessions, whereas Jaber reported a GEE treatment coefficient 1 month after session 3; Jaber provided a model-based standard error for that coefficient but not arm-level change means and variances compatible with the LIGHT analysis. Pooling would therefore have combined different treatment exposures, time points, and estimands. These outcome-specific incompatibilities are detailed in the compatibility panel in Supplementary Table S5.

### 3.4. Sexual Function, Quality of Life, and Satisfaction

Sexual-function, quality-of-life, or satisfaction outcomes were reported by 23 study families, representing approximately 976 breast cancer survivors across the largest relevant denominators; the number contributing to individual instruments and time points was smaller.

Neither sham-controlled CO_2_ trial showed a laser-specific gain in overall sexual function. LIGHT’s between-group difference in FSFI change at 6 months was 2.8 points (95% CI −1.0 to 6.5; *p* = 0.15) [6], and Jaber found no significant time or treatment effect on total or domain FSFI scores after three sessions [7]. In LARF/EPMLARF, FSFI improved over time across the CO_2_, radiofrequency, and promestriene arms, but the group-by-time interaction fell short of significance (*p* = 0.271) [8]. Gold’s randomized Er:YAG trial did not detect between-group differences in the prespecified sexual-health or quality-of-life domains; at 3 months, *p* = 0.390 for EORTC SHQ-C22 sexual pain, *p* = 0.388 for sexual satisfaction, and *p* = 0.677 for EORTC QLQ-BR45 sexual functioning [5]. No study compared CO_2_ and Er:YAG head-to-head on sexual function.

Uncontrolled CO_2_ cohorts routinely reported short-term gains: the Ohio State cohort’s mean FSFI rose 9.67 points (95% CI 7.27 to 12.08) at 4 weeks [22], and Salvatore’s rose from 19.3 to 26.8 by 20 weeks [25,26]. Longer term, the picture was less clean, Ohio State FSFI and FSDS-R scores stayed improved at 2 years with no further change between years 1 and 2 (mean change −0.83, 95% CI −3.07 to 2.38; *p* = 0.794) [20], whereas Favier found no lasting change in FSFI or SF-12, with only 41% of the cohort satisfied past 24 months [23]. Veron reported FSFI improvement that persisted but faded by a median of 19 months [24]. On the Er:YAG side, Okui’s non-randomized comparison favored combined Er:YAG plus Nd:YAG over Er:YAG alone at 24 months, though the estimate carries a serious risk of confounding and selection bias [17]. Full modality-labelled results appear in Supplementary Table S6.

### 3.5. Vaginal Health, Urinary Outcomes, and Tissue Findings

Twenty-two study families, representing approximately 910 breast cancer survivors across the largest relevant denominators, contributed at least one vaginal-health, urinary, or tissue outcome; outcome-specific denominators varied substantially.

Objective and clinician-rated measures showed no consistent comparative pattern either. Within the CO_2_ evidence, LIGHT found no between-group difference in VHI change (1.4 points, 95% CI −0.6 to 3.5; *p* = 0.17), nor in pH, maturation index, epithelial thickness, or elasticity [6]. Jaber, by contrast, reported CO_2_-associated gains in VHI (GEE coefficient 2.26 ± 0.50; *p* < 0.001), hydration (3.24 ± 1.13; *p* = 0.004), and pH (−0.31 ± 0.14; *p* = 0.021), though several individual symptoms actually favored sham [7]. In LARF/EPMLARF, VHI improved across the CO_2_, radiofrequency, and promestriene arms with no group-by-time interaction through 10 months (*p* = 0.320) [8]. In the Er:YAG trial, baseline VHI was lower in the laser group (median 9, IQR 8–13) than in the hyaluronic-acid group (13, 10–16; *p* = 0.031). Both groups improved (*p* = 0.001), but the 3-month comparison did not detect a difference (*p* = 0.232); arm-level follow-up values and a contrast confidence interval were not reported [5].

Urinary data were thin and inconsistent. Gold did not detect a between-group difference in pelvic-floor outcomes at 3 months (*p* = 0.650 for the total pelvic-floor score) [5]. In LARF/EPMLARF, urinary symptom frequency showed a group-by-time interaction favoring the two energy-based arms (*p* = 0.040), but I-QOL did not (*p* = 0.182) [8]. UDI-6 improved at 4 weeks in the Ohio State cohort but drifted back toward baseline by 2 years [20,22], and the urinary quality-of-life gain Veron reported at 6 months did not hold to the end of follow-up [24].

Most single-arm CO_2_ and Er:YAG cohorts reported short-term rises in VHI or related indices, but being uncontrolled, none of this establishes a modality-specific effect. Controlled tissue evidence was limited to CO_2_: the LARF/EPMLARF biopsy substudies found no post-treatment histological differences among CO_2_ laser, radiofrequency, and promestriene, and no structural damage in any arm [15,16]. In an uncontrolled CO_2_ histology cohort, elastin and collagen type III indices rose after five sessions while epithelial morphology, total collagen, and collagen type I stayed unchanged [19]. These findings are consistent with possible stromal remodeling but fall short of demonstrating clinical regeneration. Modality-labelled vaginal, urinary, and tissue results are provided in Supplementary Table S7.

### 3.6. Durability of Response

Ten study families, with approximately 614 participants at baseline or in the principal analytic samples, reported at least one outcome at 6 months or later; the numbers retained at extended follow-up were substantially smaller.

Under this operational definition, no controlled durability data extend beyond approximately 10 months. All evidence at ≥12 months came from uncontrolled cohorts with substantial attrition or selective retention and is described as extended follow-up rather than evidence of established long-term efficacy. Some symptom and sexual-function gains persisted, but fading effects and requests for retreatment were common. The Ohio State cohort’s combined vaginal-symptom and sexual-function measures stayed broadly stable through 2 years, though only 33 of the 67 enrolled participants contributed data at that point [20,21,22]. In Veron, FSFI remained above baseline at a median of 19 months, but urinary quality of life drifted back toward baseline and 58% of participants requested a maintenance session [24]. Favier found select symptoms still improved past 24 months, but not FSFI or SF-12, with 38% of participants dissatisfied [23].

Gambacciani and Levancini’s Er:YAG cohort showed dryness, dyspareunia, and VHI effects waning to the point of no longer differing from baseline at 18 months [30,31]. Okui found pain and FSFI improvement held to 24 months only in the combined Er:YAG-plus-Nd:YAG group, while VHI regressed toward atrophic values in both arms [17]. Because these are uncontrolled, selectively retained cohorts, they cannot determine whether maintenance treatment is actually effective. Durability findings are summarized in Supplementary Table S8. No included study evaluated a prespecified retreatment protocol; reports of maintenance requests or additional sessions were observational and did not establish retreatment efficacy.

### 3.7. Safety and Tolerability

Safety or tolerability was mentioned across all 28 study families, representing approximately 1190 participants in the largest relevant randomized, treated, or matched samples; however, event-specific denominators and surveillance methods were often incompletely reported.

No included study reported a serious treatment-related adverse event, though sample sizes and follow-up were too limited to exclude rare or delayed harms. In LIGHT, related events stayed mild to moderate, none reached severe, and serum estradiol remained at menopausal levels [6]. In Jaber, minor spotting or vaginal discomfort occurred in 5 of 19 laser-treated and 7 of 15 sham-treated participants (*p* = 0.288), with one cystitis case requiring antibiotics in each arm [7]. The Ohio State cohort reported grade 1–2 discharge and dryness but nothing grade 3 or higher through 2 years [20,22].

Across the CO_2_, Er:YAG, and other laser studies, the events reported most often were transient procedural pain or heat, spotting or mild bleeding, discharge, edema, candidiasis, and cystitis [8,17,29,33,34,35,36,37,38,39,40,41,42,43]. The CO_2_ LARF/EPMLARF biopsy studies found neither burns nor clinical or histological tissue damage [15,16]. Favier reported one case of atypical endometrial hyperplasia identified three years after treatment in a participant using hormone replacement therapy, with causality never established [23]. Event definitions, active surveillance, and denominators varied enough between reports that several offered only general statements of tolerability. Taken together, the evidence is insufficient to compare the safety of CO_2_ and Er:YAG or to exclude rare or delayed harms (Supplementary Table S8). Study-level adverse-event ascertainment, reported events, severity, and limitations are presented in Supplementary Table S12.

### 3.8. Risk of Bias in Randomized Studies

All five randomized studies (three CO_2_, two Er:YAG) were assessed with RoB 2 for their principal patient-reported or primary efficacy outcomes. LIGHT and Jaber, both CO_2_ trials, raised some concerns overall, mainly complete-case analyses after attrition and, for Jaber, incomplete reporting on allocation concealment and unverified masking. Gold’s Er:YAG trial was judged at high outcome-specific risk of bias: allocation concealment and assessor masking were not reported, one ineligible participant was excluded after randomization, baseline VHI differed between groups, and participants were aware of treatment while reporting subjective outcomes. Core symptom and VHI denominators were retained, but response counts varied for some questionnaire domains. The CO_2_ LARF/EPMLARF trial and the Fidecicchi pilot assessed from a detailed abstract were also judged at high overall risk of bias, driven by substantial missing data in LARF/EPMLARF and insufficient methodological detail for Fidecicchi (Figure 3; Supplementary Table S2).

### 3.9. Risk of Bias in Non-Randomized Comparative Evidence

Okui was the only non-randomized study directly comparing two laser strategies, so it alone was assessed with ROBINS-I, and it was judged at serious overall risk of bias. Treatment strategy was assigned by calendar period, leaving residual and secular confounding even after propensity-score matching; participants with less than two years of follow-up or missing data were excluded post hoc and unevenly between groups, and the main extended-follow-up pain outcome was self-reported in an unblinded retrospective analysis (Supplementary Table S3). Longitudinal uncontrolled cohorts and observational subgroup studies were appraised separately with the JBI cohort checklist [13]. Domain-level ROBINS-I judgments were serious for confounding, serious for participant selection, low for intervention classification, moderate for deviations from intended interventions, serious for missing data, moderate for outcome measurement, and moderate for selection of the reported result; the overall judgment was serious.

### 3.10. Risk of Bias in Observational Cohort Evidence

The remaining 22 observational study families were appraised with the JBI cohort checklist (Supplementary Table S4). Laser exposure protocols and outcome measurement were generally reported adequately, but the designs themselves, uncontrolled or built around indirect subgroup comparisons, limited what the evidence could show. Only Siliquini used a matching strategy to address confounding; the other 21 cohorts applied no analytical strategy for it. Follow-up completeness was inadequate in seven cohorts and unclear in four, and where follow-up was incomplete, studies typically fell back on complete-case analysis or did not describe how it was handled. Reporting uncertainty was greatest for the detailed-abstract-only Lami report and the registry-results-only AFLT study. No aggregate JBI score or numerical exclusion threshold was applied.

### 3.11. Certainty of Evidence

Using GRADE, the certainty of the sham-controlled CO_2_ evidence was rated low for dyspareunia and overall sexual function, and very low for vaginal dryness and VHI. For the separately defined Er:YAG-versus-hyaluronic-acid question, certainty was very low for vaginal dryness, dyspareunia, EORTC SHQ-C22 sexual pain, EORTC QLQ-BR45 sexual functioning, and VHI. The Er:YAG ratings were downgraded one level for serious risk of bias and two levels for very serious imprecision. The single trial analyzed 43 participants and reported arm-level medians, interquartile ranges, and between-group *p*-values, but no between-group effect estimates with 95% confidence intervals; clinically important benefit or harm therefore could not be excluded. Inconsistency was not assessable and was not used to downgrade. The active comparator itself was neither an exclusion criterion nor a reason for downgrading, and nonsignificant *p*-values were not interpreted as evidence of equivalence. These estimates address comparative effectiveness against hyaluronic acid and do not establish an Er:YAG-specific effect relative to sham or no treatment. Certainty for serious adverse events across the randomized evidence was also very low. Supplementary Table S9 provides the contrast-specific summary of findings and the reasoning behind each judgment. No outcome reached moderate or high certainty.

No secondary outcome met the prespecified bar for pooling. FSFI and VHI were each measured in more than one comparative study, but the trials varied in laser modality, regimen, comparator, follow-up, and reported estimand, and Jaber reported model coefficients rather than arm-level change variances that could be combined. Separate CO_2_ and Er:YAG meta-analyses were likewise ruled out, since fewer than two clinically compatible comparisons existed per modality-outcome pair. Consequently, no pooled estimate, heterogeneity statistic, subgroup analysis, sensitivity analysis, or small-study-effect assessment was undertaken.

### 3.12. Sensitivity Analysis Restricted to Full-Text Peer-Reviewed Evidence

A post hoc qualitative sensitivity analysis excluded outcome results available to the review team only as detailed abstracts and excluded the registry-results-only AFLT study. Full-text findings from linked VagLaser and Quick/Ohio State publications were retained, while abstract-only outcome components were removed. The principal interpretation was unchanged: the full-text sham-controlled CO_2_ evidence still showed no reproducible laser-specific benefit for dyspareunia or overall sexual function; the daily dryness signal remained isolated; the full-text Er:YAG-versus-hyaluronic-acid trial did not demonstrate superiority; and no direct CO_2_-versus-Er:YAG comparison was available. Uncertainty regarding durability and uncommon or delayed harms also remained. Because no outcome contained two clinically and statistically compatible full-text comparisons, a quantitative sensitivity meta-analysis was not possible.

## 4. Discussion

### 4.1. Principal Findings

This review identified 28 study families evaluating vaginal laser therapy for GSM in breast cancer survivors, and the clearest pattern to emerge is a contrast: uncontrolled cohorts consistently reported favorable within-group change, while randomized comparative studies told a less consistent story. Both sham-controlled trials, each testing fractional CO_2_, failed to show a reproducible laser-specific benefit for dyspareunia or overall sexual function; one did find daily dryness and a few objective measures favoring CO_2_, but not intercourse dryness or dyspareunia [6,7]. Active-comparator trials of CO_2_ and Er:YAG generally showed improvement across all treatment groups, with no consistent sign that laser outperformed radiofrequency, promestriene, hyaluronic acid, or other active strategies [5,8].

None of this means participants experienced no improvement, only that symptom improvement after treatment cannot automatically be attributed to a laser-specific biological effect. Co-interventions, repeated clinical contact, expectation effects, regression to the mean, and the naturally fluctuating course of GSM can all drive change in both laser and comparator arms alike. Keeping within-group improvement and comparative efficacy conceptually separate is, in our view, central to interpreting the present evidence correctly.

### 4.2. CO_2_ Laser Evidence

CO_2_ dominates this evidence base and supplied the only sham-controlled data available. In LIGHT, five monthly fractional CO_2_ sessions performed no better than sham on FSFI, dyspareunia, VHI, pH, maturation, epithelial thickness, or elasticity at 6 months [6]. Jaber’s picture after three sessions was more mixed: daily dryness, VHI, hydration, and pH favored laser, but the co-primary outcomes, intercourse dryness and dyspareunia, did not [7]. Differences in schedule, outcome definition, follow-up, and statistical approach may explain some of this divergence, but it still leaves a CO_2_-specific effect difficult to reproduce.

LARF/EPMLARF further illustrates how much the comparator matters: CO_2_, radiofrequency, and promestriene all tracked toward improvement over time, but none pulled ahead on dryness, dyspareunia, FSFI, or VHI through 10 months [8]. Its histological substudies found no structural advantage for CO_2_ over the active comparators, and no tissue damage in any arm [15,16]. Improvement after CO_2_ treatment, in other words, was not consistently distinguishable from improvement produced by other interventions.

Uncontrolled CO_2_ cohorts, unsurprisingly, reported the largest short-term changes in dryness, dyspareunia, sexual function, and vaginal-health scores, and some of this held up among retained participants, the Ohio State cohort’s combined symptom and sexual-function measures, for instance, through 2 years [20,21,22,23,24,25,26,27,28,32,33,34,36,39,41,42,43]. Attrition, however, was substantial, and extended-follow-up findings were far from uniform: Favier found select symptoms still improved past 24 months but not FSFI or SF-12, with limited satisfaction overall [23], while Veron saw sexual and urinary gains fade along with frequent requests for maintenance treatment [24]. Most CO_2_ protocols in this review were fractional or microablative; the exception was one small abstract-only study whose authors described their protocol as ‘non-ablative,’ though the available report gave too little technical, numerical, or comparative detail to verify that descriptor or analyze it as a separate CO_2_ subgroup [39].

### 4.3. Er:YAG Laser Evidence

The Er:YAG evidence base is smaller, and no sham-controlled trial exists for it. Gold reported within-group improvement, but the trial did not detect superiority of Er:YAG over hyaluronic acid for dryness, dyspareunia, sexual health, VHI, or pelvic-floor outcomes; the small sample and incomplete effect reporting leave clinically important differences uncertain [5]. Fidecicchi reported a greater short-term reduction in superficial dyspareunia with a vestibular and introital hyperstack protocol versus standard Er:YAG SMOOTH treatment, but the available abstract gave no arm-level means, variance estimates, or enough methodological detail for a precise effect estimate [18].

Okui compared Er:YAG alone with combined Er:YAG plus Nd:YAG and found more durable improvement in superficial vulvar pain and FSFI with the combined approach at 24 months, an intriguing result, since it hints that treating the vulvar pain component specifically may influence dyspareunia [17]. Treatment allocation, however, tracked calendar period, complete-case selection during extended follow-up differed between groups, and confounding persisted despite propensity-score matching. This finding is best regarded as hypothesis-generating rather than as evidence that the combined strategy is superior.

The Arêas, Gambacciani/Levancini, Mothes, and Ferreira cohorts each reported short-term within-group improvement after Er:YAG treatment [29,30,31,35,37]. The longest available follow-up showed the effect fading progressively; dryness, dyspareunia, and VHI stayed improved through 12 months but lost statistical significance by 18 [30,31]. Overall, Er:YAG appears feasible and may relieve symptoms in the short term, but this evidence is dominated by small uncontrolled studies or active-comparator designs and cannot establish a laser-specific effect.

Across the evidence hierarchy, laser-specific efficacy can be inferred only from sham-controlled trials. Gold et al. informs active-comparator effectiveness, whereas the Fidecicchi protocol comparison, available to the review team only as a detailed abstract and judged at high RoB 2 risk, and the retrospective Okui comparison, judged at serious ROBINS-I risk, are hypothesis-generating. Neither establishes modality-level efficacy or superiority.

### 4.4. Comparative Interpretation of CO_2_ and Er:YAG

CO_2_ and Er:YAG should not be interpreted as interchangeable interventions. Fractional or microablative CO_2_ protocols create microscopic ablative columns surrounded by thermal effect, whereas Er:YAG SMOOTH protocols are mostly non-ablative or subablative and deliver more superficial thermal energy; treatment schedules, energy settings, anatomical targets, and session counts also varied within each modality. These are genuine technical differences, and they justify analyzing the two modalities separately, but they do not demonstrate that one is clinically superior.

CO_2_’s apparently larger volume of positive evidence mostly reflects a larger number of cohorts rather than stronger comparative effects, and the promising-looking Er:YAG results come chiefly from smaller active-comparator or uncontrolled studies. No study in this review compared CO_2_ with Er:YAG directly in breast cancer survivors, so any cross-study contrast would be confounded by differences in population, endocrine-therapy exposure, symptom severity, protocol, comparator, outcome scale, and follow-up. Direct comparative evidence is simply lacking, and neither superiority nor equivalence between the modalities can be established on this basis.

Evidence for other laser systems is too limited to support broader inference. The dual-wavelength diode and other solid-state vaginal-laser cohorts reported short-term within-group improvement, but each represented a small treated cohort or subgroup with no untreated comparator [38,40], and these technologies should remain analytically separate from both CO_2_ and Er:YAG.

### 4.5. Durability and Safety

Durability remains uncertain for both principal modalities. Controlled evidence extends only to approximately 10 months, whereas all extended follow-up at ≥12 months comes from uncontrolled cohorts marked by substantial attrition or selected retention of responders. These observations should not be interpreted as established long-term efficacy. CO_2_ studies reported persistence of some symptom or sexual-function outcomes through 18–24 months, but attenuation, dissatisfaction, and requests for retreatment were common [20,23,24]. The best-described Er:YAG cohort lost statistical significance by 18 months [30,31], and while the favorable 24-month Okui result is appealing, it carries a serious risk of bias [17]. The available data do not define an optimal maintenance schedule or establish whether retreatment is effective.

No serious treatment-related adverse event was identified, and both CO_2_ and Er:YAG were generally described as tolerable procedures, with reported events limited mostly to transient pain or heat, spotting, mild bleeding, discharge, edema, candidiasis, or cystitis [6,7,8,17,29,41]. Most studies, however, were small, follow-up was incomplete, adverse-event definitions were inconsistent, and active surveillance was often not prespecified, so an absence of observed serious events should not be read as establishing safety over extended follow-up or as evidence that the two modalities are equally safe. Rare and delayed harms remain insufficiently evaluated.

### 4.6. Clinical Implications and Future Research

Sham-controlled CO_2_ certainty ranges from low to very low, and Er:YAG-versus-hyaluronic-acid evidence is of very-low certainty. The latter addresses comparative effectiveness against an active nonhormonal treatment and cannot isolate a laser-specific effect; no sham-controlled Er:YAG evidence is available. Vaginal laser should therefore not be presented as an established modality-specific treatment with proven superiority over sham or active nonhormonal options. In clinical practice, this means being transparent about the uncertainty in laser-specific efficacy, durability, need for maintenance, and uncommon or delayed harms. Patient preference and the real limitations of available hormonal and nonhormonal options remain relevant, particularly for women receiving aromatase inhibitors. However, endocrine-therapy status remains a major evidence gap: the aromatase-inhibitor-restricted LIGHT trial cannot compare endocrine regimens, the few subgroup analyses were small, unadjusted, or abstract-only, and the only explicitly reported aromatase-inhibitor-plus-LHRH subgroup comprised six women without separate outcome estimates [6,24,25,26,39,42]. The evidence therefore cannot support different counseling or expected response according to aromatase-inhibitor use, tamoxifen, ovarian suppression, or no current endocrine therapy.

What this field needs are modality-specific trials for CO_2_ and Er:YAG that use standardized protocols and credible sham controls, with core outcomes covering daily dryness, intercourse dryness, superficial and deep dyspareunia separately, validated sexual-function measures, VHI or another objective index, urinary symptoms, quality of life, and systematically collected adverse events. Follow-up should extend beyond 12 months, report maintenance or retreatment explicitly, and build in strategies that minimize attrition. A direct CO_2_-versus-Er:YAG trial would be genuinely valuable, provided its parameters are prespecified and it is powered for clinically important between-group differences. Stratifying by aromatase inhibitor use, tamoxifen, ovarian suppression, natural menopause, and baseline symptom phenotype could help clarify whether treatment response differs across these clinically relevant subgroups.

### 4.7. Strengths and Limitations

This review’s strengths lie in a broad database and registry search, careful linkage of multiple reports to their underlying study families, a clean separation of comparative efficacy from durability and safety, modality-specific synthesis, and risk-of-bias assessment matched to each design. Distinguishing sham-controlled evidence from active-comparator and uncontrolled evidence throughout also reduces the risk of mistaking within-group change for genuine treatment efficacy.

The review’s limitations, in turn, mostly trace back to the underlying evidence itself. Outcome definitions, scales, protocols, comparators, and follow-up times were highly heterogeneous, and compatible change-score variances were rarely reported. Several reports existed only as detailed abstracts or registry results, some cohorts may overlap, extended-follow-up analyses often relied on complete cases, and adverse-event surveillance was described inconsistently. Together, these issues precluded meta-analysis and reduced confidence in modality-specific and subgroup conclusions. Publication and selective-reporting bias also cannot be excluded, since too few comparable studies existed to test for it formally.

PROSPERO registration occurred after title screening had started, although the protocol had been finalized before screening and no substantive changes were made to eligibility, outcomes, modality grouping, or synthesis rules. In addition, several outcome results were available only as detailed abstracts or registry results. The planned mean differences, standardized mean differences, and risk ratios could not be generated consistently because compatible between-group estimates, variance data, and common time points were frequently unavailable. Narrative synthesis was the prespecified alternative when pooling was not methodologically defensible, but the limited implementation of the planned effect measures remains a constraint on quantitative interpretation. The full-text-only sensitivity analysis did not change the central conclusions.

## 5. Conclusions

Vaginal laser therapy in breast cancer survivors shows short-term improvement across many uncontrolled studies, but comparative evidence stops short of a consistent laser-specific benefit on the outcomes that matter most to patients. CO_2_ has the larger evidence base because it includes more studies and the only sham-controlled trials; this should not be interpreted as evidence of greater efficacy. The Er:YAG-versus-hyaluronic-acid comparison is supported by very-low-certainty evidence, while the remaining Er:YAG results rest mainly on uncontrolled or protocol-comparison studies, with signs of fading benefit over extended follow-up. No direct comparison supports either superiority or equivalence between CO_2_ and Er:YAG. Each modality was generally described as tolerable in the short term, but cross-modality safety comparisons are not possible, and durability, maintenance strategies, and uncommon or delayed harms remain open questions. Well-powered, modality-specific trials are needed, including credible sham-controlled trials and direct head-to-head comparisons with standardized outcomes and adequately powered extended follow-up beyond 12 months. Future trials should prioritize a core outcome set comprising daily and intercourse dryness, superficial and deep dyspareunia, validated sexual-function measures, an objective vaginal-health measure, urinary symptoms, quality of life, and systematically ascertained adverse events. Prespecified analyses should prioritize women receiving aromatase inhibitors, tamoxifen, ovarian suppression, and no current endocrine therapy.

## Figures and Tables

**Figure 1:**
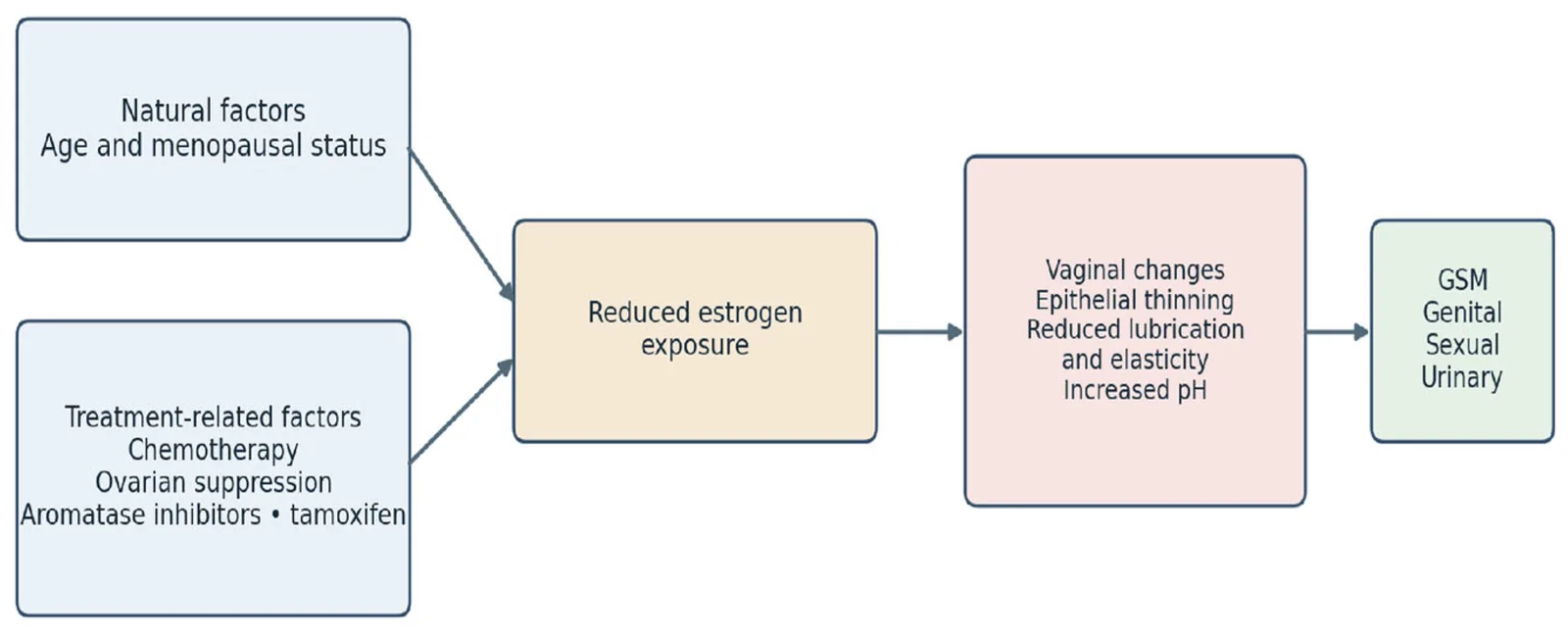
Interaction of age-related and treatment-related factors in the development of genitourinary syndrome of menopause among breast cancer survivors.

**Figure 2:**
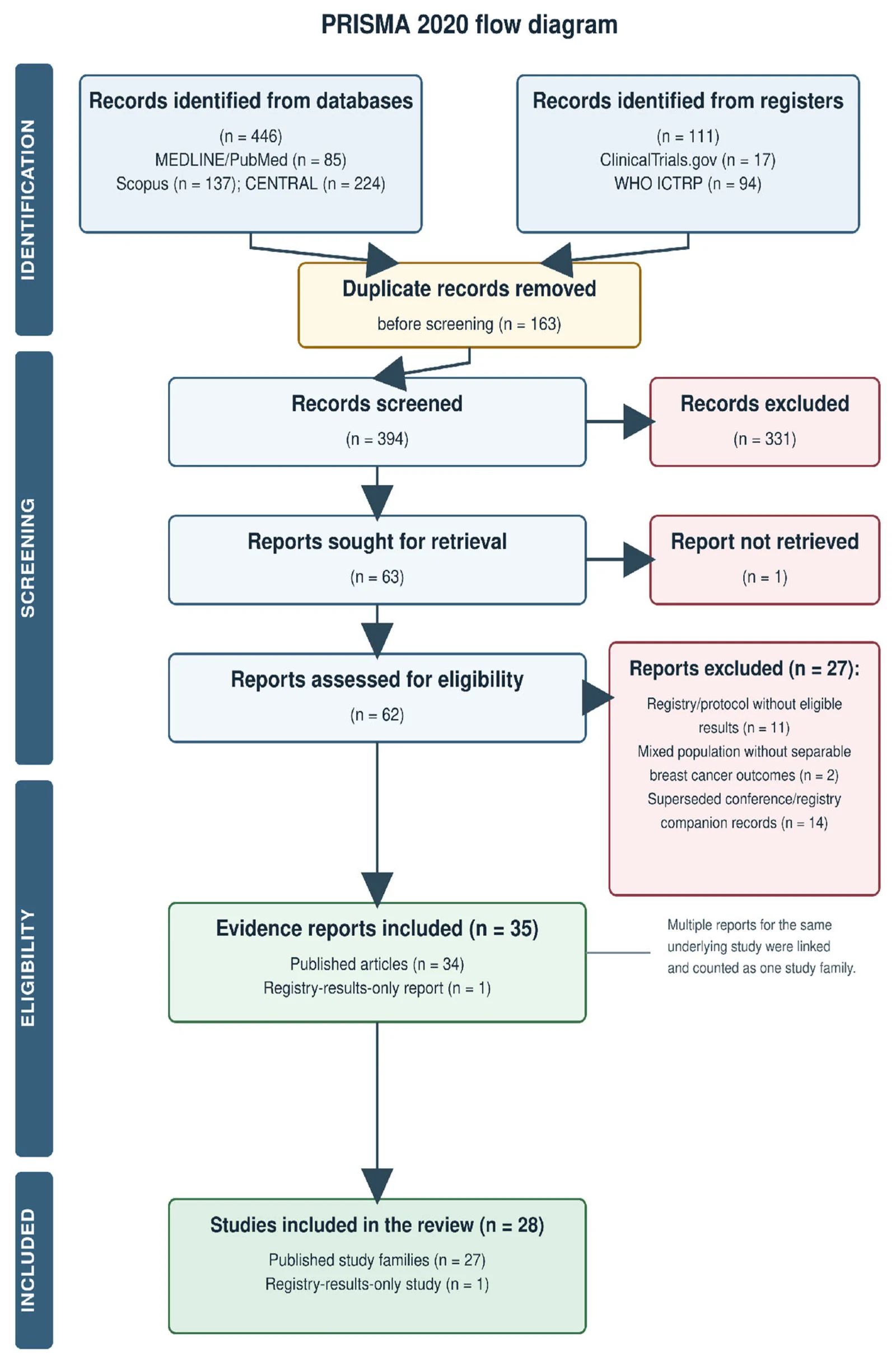
PRISMA 2020 flow diagram of study identification and selection. Multiple publications, conference abstracts, and registry records relating to the same underlying study were linked to avoid double counting. The registry-results-only study was retained as additional unpublished evidence. Reasons for exclusion were coded at the full-text stage; the 331 title/abstract exclusions were not categorized individually.

**Figure 3:**
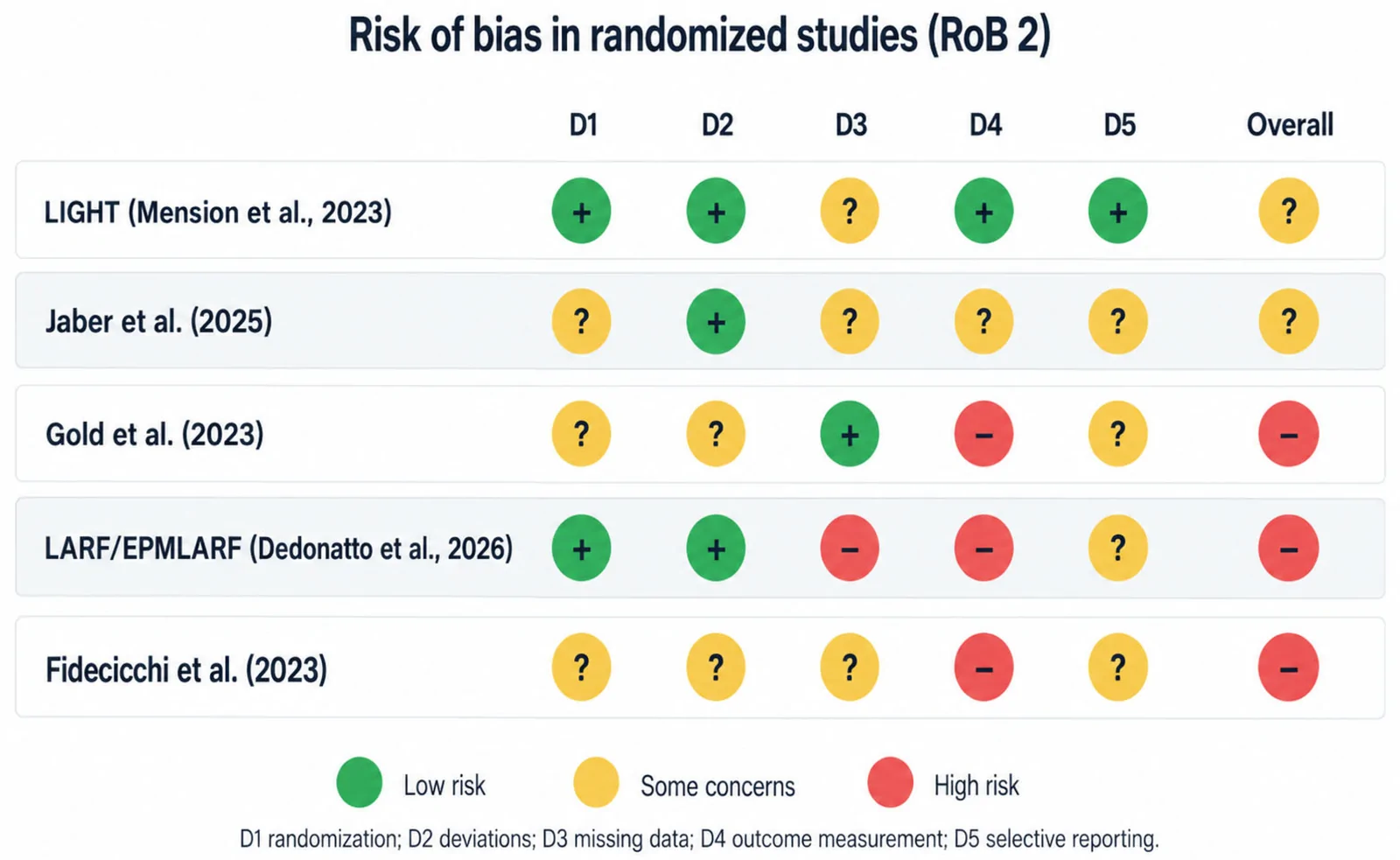
Risk-of-bias judgments for randomized studies using the Cochrane RoB 2 tool. Judgments apply to the specified primary or patient-reported efficacy outcome and time point [5,6,7,8,15,16,18].

**Table 1:** Characteristics of included studies.

Study; Country	Design and Sample	Population and Cancer/Endocrine Therapy	Laser Intervention	Comparator	Follow-Up	Outcomes
**LIGHT** [6] ** Spain**	Prospective double-blind sham-controlled parallel RCT N: 84 randomized; 72 analyzed (35 CO_2_, 37 sham)	Breast-cancer survivors with GSM receiving aromatase inhibitors Aromatase inhibitors; both groups also received moisturizers and vaginal vibrator stimulation	Fractional CO_2_; 5 monthly sessions	Sham laser, 5 monthly sessions	6 months	FSFI primary; dyspareunia, dryness, VHI, VMI, pH, urinary and safety outcomes
**Jaber sham-controlled RCT** [7] ** Israel**	Prospective randomized single-blind sham-controlled trial with subsequent open phase N: 34 completed comparative phase (19 CO_2_, 15 sham); 2 sham withdrawals reported	Breast-cancer survivors with GSM and moderate/severe symptoms Mixed prior/current anti-estrogen exposure reported	Fractional CO_2_; 3 sessions every 3–4 weeks, with optional extension to 6	Sham procedure for 3 sessions	1 month after session 3; open-phase follow-up after session 6	Co-primary VAS intercourse dryness and dyspareunia; FSFI, VHI and safety
**Gold laser versus hyaluronic acid** [5] ** Austria**	Single-center randomized active-comparator trial N: 43 analyzed after 1 post-randomization exclusion (22 laser, 21 hyaluronic acid)	Women with previous breast cancer and symptomatic urogenital atrophy Antihormonal therapy common; AI and tamoxifen reported	Intravaginal Er:YAG; 2 applications 1 month apart	Hyaluronic-acid suppositories daily for 10 days, then every third day through 3 months	3 months	VHI primary; symptom bother, pelvic-floor symptoms, QoL, sexual health, satisfaction and adverse events
**LARF/EPMLARF arm 2** [8,15,16] ** Brazil**	Multi-arm randomized active-comparator trial with companion histology reports N: Main 10-month analysis: 58 completers (21 CO_2_, 19 RF, 18 promestriene); companion 4-month histology reports: 70 randomized	Postmenopausal breast-cancer survivors with GSM receiving adjuvant endocrine therapy All receiving adjuvant endocrine therapy; tamoxifen and aromatase inhibitors represented	Fractional CO_2_; 3 monthly sessions	Microablative radiofrequency, 3 monthly sessions; or topical promestriene	4 months in histology reports; 10 months in main clinical report	GSM symptoms, VHI, sexual and urinary outcomes, satisfaction, adverse events, vaginal/vulvar histology
**Okui two-strategy laser cohort** [17] ** Japan**	Retrospective non-randomized comparative cohort with propensity-score matching N: 256 eligible; propensity-matched analysis 102 VEL+Nd:YAG versus 102 VEL	Breast-cancer survivors with GSM and superficial vulvar pain/dyspareunia Tamoxifen and aromatase-inhibitor exposure reported	Er:YAG vaginal plus Nd:YAG vulvar treatment; 3 sessions every 30 days	Er:YAG vaginal laser alone, 3 sessions every 30 days	1, 3, 6, 12 and 24 months	Dyspareunia/vulvar pain VAS, swab pain mapping, FSFI and adverse events
**Fidecicchi hyperstacking pilot** [18] ** Italy/Argentina**	Prospective randomized active-comparator pilot N: 68 total (34 standard VEL, 34 hyperstack)	Postmenopausal breast-cancer survivors with GSM and superficial dyspareunia Breast-cancer survivor population; detailed therapy distribution unavailable in abstract	Standard Er:YAG SMOOTH; 3 sessions at 30-day intervals	Modified Er:YAG hyperstack treatment of vestibulum/introitus, 3 sessions	1 and 3 months after last session	Superficial dyspareunia VAS
**VagLaser dose/histology programme** [9,19] ** Denmark**	Prospective single-center dose-determining cohort with companion histology study N: 30 enrolled; 27 completed five sessions in dose report; 26 paired-biopsy participants in histology report	Breast-cancer survivors with symptomatic GSM receiving endocrine therapy SERM or aromatase-inhibitor adjuvant therapy	Fractional microablative CO_2_; 5 monthly sessions	No comparator	Assessments after sessions 3, 4 and 5; histology 4–6 weeks after session 5	Dryness VAS primary; pain/itching/soreness, FSFI, urinary measures, VHI and vaginal histology
**Quick/Ohio State cohort** [20,21,22] ** United States**	Prospective single-arm feasibility cohort with 12- and 24-month follow-up reports N: 67 enrolled; 64 treated; 59 completed 4-week follow-up; 39 at 12 months; 33 at 24 months	Breast-cancer survivors with dyspareunia and/or vaginal dryness Most participants receiving endocrine therapy; AI and tamoxifen represented	Fractional CO_2_; 3 treatments at 30-day intervals	No comparator	4 weeks, 12 months and 24 months	Vaginal symptom VAS, FSFI, FSDS-R, UDI, objective vaginal measures and adverse events
**Favier 24-month cohort** [23] ** France**	Retrospective single-center mixed cohort with breast-cancer subgroup N: 46 total; separately reported breast-cancer subgroup *n* = 26	Postmenopausal women treated for GSM; breast-cancer subgroup separately analyzed In overall cohort 9 remained on antihormone therapy; subgroup analyses by breast-cancer and endocrine treatment	Fractional CO_2_; 3 sessions 4–6 weeks apart	No comparator	At least 24 months	Satisfaction primary; GSM symptoms, FSFI, SF-12, local-treatment use and adverse events
**Veron extended-follow-up cohort** [24] ** France**	Prospective single-center single-arm cohort N: 46 included; 45 completed all sessions; 42 at 6 months; 37 at end of study	Postmenopausal women with breast cancer and GSM/VVA 36/46 receiving endocrine therapy: tamoxifen 6, AI 24, AI plus LHRH agonist 6	Fractional microablative CO_2_; 3 monthly sessions	No comparator	6 months and approximately 18 months; median follow-up 19 months	FSFI, Ditrovie urinary QoL, SF-12, vaginal pH, Pap-smear maturation pattern, adverse events
**Salvatore five-session pilot** [25,26] ** Italy**	Prospective single-arm cohort with comparison by current versus previous endocrine therapy N: 40 enrolled and completed 20-week follow-up	Women with previous breast cancer and symptomatic VVA All had previous or current endocrine therapy; subgrouped by current versus past treatment	Microablative fractional CO_2_; 5 applications every 4 weeks	No untreated comparator; internal endocrine-status subgroup comparison	20 weeks from first treatment	Symptom VAS, VHI, FSFI, SF-12, satisfaction and adverse events
**Siliquini BCS/healthy comparison** [27] ** Italy**	Retrospective matched comparative cohort; all participants received laser N: 135 total: 45 breast-cancer survivors and 90 age/BMI-matched women without breast cancer	Postmenopausal women with GSM; breast-cancer subgroup separately reported BCS included chemotherapy and endocrine-therapy exposure	Fractional microablative CO_2_; vaginal and vulvar application, 3 sessions every 30 days	Women without breast cancer receiving the same laser; not an untreated efficacy control	After each session and 3, 6 and 12 months after treatment	Dyspareunia and dryness VAS, VHI, VVHI, procedural pain and adverse events
**LAAVA pilot** [28] ** Australia**	Prospective single-arm investigator-initiated pilot N: 26 enrolled; all received 3 treatments	Postmenopausal women with stage I-III early breast cancer and symptomatic VVA 25/26 had received anti-estrogen therapy	Fractional CO_2_; 3 treatments 4 weeks apart	No comparator	Baseline, 4, 8 and 12 weeks	VVA symptom VAS, FSFI, patient-reported symptom/sexual/QoL improvement
**Arêas vaginal Er:YAG cohort** [29] ** Brazil**	Open prospective single-arm therapeutic intervention N: 24 analyzed; one pre-treatment dropout reported	Postmenopausal women with prior breast cancer, dryness and/or dyspareunia No vaginal hormone therapy for at least 6 months; chemotherapy/endocrine histories reported	Er:YAG; 3 sessions at 30-day intervals	No comparator	One month after the third session	VHI and components, SPEQ sexual function and genitourinary symptoms, complications
**Gambacciani/Levancini Er:YAG pilot** [30,31] ** Italy**	Prospective single-arm pilot N: 43 treated; 37 valid completers	Postmenopausal breast-cancer survivors with severe GSM Successful breast-cancer treatment; no concomitant hormonal/local vaginal treatment	Nonablative Er:YAG SMOOTH; 3 applications every 30 days	No comparator	1, 3, 6, 12 and 18 months	Dryness and dyspareunia VAS, VHI and adverse events
**Pieralli dyspareunia cohort** [32] ** Italy**	Prospective descriptive single-arm cohort N: 50 included and completed treatment; VHI measured in first 36	Breast-cancer survivors with VVA-related dyspareunia 22/50 on adjuvant therapy: tamoxifen 20 and AI 2	Fractional microablative CO_2_; 3 sessions	No comparator	30 days after treatment and telephone follow-up mean 11 months	Dyspareunia VAS, VHI, satisfaction and complications
**Pagano 2016 cohort** [33] ** Italy**	Retrospective observational single-arm cohort N: 26 women	Hormone-receptor-positive breast cancer with therapy-related menopause and VVA Chemotherapy and/or hormonal therapy	Fractional microablative CO_2_; 3 cycles every 30–40 days	No comparator	30 days after third cycle	VVA symptom VAS, procedure discomfort and adverse reactions
**Pagano 2018 larger cohort** [34] ** Italy**	Retrospective observational single-arm cohort N: 82 treated; 77 completed all 3 cycles	Breast-cancer survivors with iatrogenic VVA after failure of nonestrogenic local treatments 52 received chemotherapy, 61 hormonal therapy, 42 both; AI and tamoxifen represented	Fractional microablative CO_2_; 3 cycles every 30–40 days	No comparator	30 days after third cycle	Sexual and genitourinary symptom VAS, procedural discomfort and adverse effects
**Mothes dual-phase Er:YAG cohort** [35] ** Germany**	Retrospective development/performance analysis; single treated cohort N: 16 breast-cancer survivors	Postmenopausal breast-cancer survivors with residual GSM/pelvic-floor symptoms after prolapse surgery Anticancer treatment details incompletely reported	Dual-phase Er:YAG; single 10 min treatment	No comparator	Mean 8.3 weeks	VHI, vaginal pH, subjective satisfaction and complications
**Chung Korean cohort** [36] ** Republic of Korea**	Prospective single-arm cohort N: 30 enrolled; 24 completed final assessment	Breast-cancer survivors with GSM 12 prior chemotherapy; 11 tamoxifen; 1 aromatase inhibitor; 12 no endocrine therapy	Fractional CO_2_; 3 sessions 4 weeks apart	No comparator	12 weeks	Symptom VAS, VHI, satisfaction and adverse events
**Ferreira Er:YAG cohort** [37] ** Brazil**	Prospective single-arm pilot with tissue assessment N: 12 enrolled; 10 completed early assessment; 8 at 1 year	Breast-cancer survivors with GSM/VVA Anticancer-treatment history reported in full text	Er:YAG; 3 monthly sessions	No comparator	1 month after treatment and 1 year from start	VHI, symptom severity, FSFI, biopsy/cytology, satisfaction and adverse events
**Perrini dual-wavelength diode cohort** [38] ** Italy**	Prospective single-arm cohort with prespecified subgroup comparison N: 30 recruited; 29 completed; breast-cancer subgroup *n* = 13	Postmenopausal women with GSM; breast-cancer subgroup separately analyzed Breast-cancer treatment exposure reported by subgroup	Dual-wavelength diode; 3 sessions every 4 weeks	Non-breast-cancer treated subgroup; no untreated control	4 weeks after third session	VHI, VMI, dyspareunia, procedural pain and adverse events
**Lami CO_2_ cohort** [39] ** Italy**	Prospective single-arm cohort N: 26 breast-cancer survivors	Breast-cancer survivors with VVA Aromatase inhibitor, tamoxifen and no-therapy subgroups	CO_2_ described by the authors as nonablative; 3 sessions	No comparator; internal adjuvant-therapy subgroup comparison	1 month after third session	VHI, VuHI, symptom VAS, FSFI, FSDS and MENQOL
**Lubián-López SSVL cohort** [40] ** Spain**	Prospective single-arm cohort N: 38 informed; 36 consented; 30 eligible; 27 at 10 weeks; 24 at 24 weeks	Women with estrogen-dependent breast cancer and GSM Aromatase-inhibitor history reported	Nonablative solid-state vaginal laser protocol	No comparator	10 and 24 weeks	Dyspareunia, VHI, VuHI, FSFI, vaginal pH, VMI, satisfaction and adverse events
**Hersant EPIONE feasibility cohort** [41] ** France**	Prospective single-center feasibility study N: 20 breast-cancer survivors	Breast-cancer survivors with vulvovaginal atrophy Anticancer-treatment history reported	Fractional CO_2_; 2 sessions	No comparator	1, 3 and 6 months	VHI, female sexual distress and adverse events
**Gittens and Mullen endocrine subgroup** [42] ** United States**	Retrospective single-arm cohort with separately reported breast-cancer subgroup N: 25 total; breast-cancer endocrine-therapy subgroup *n* = 8	Women treated for GSM; target subgroup had breast cancer and endocrine therapy Endocrine therapy in target subgroup	Fractional CO_2_; 3 sessions 6 weeks apart	No untreated comparator	Through third treatment	FSFI, FSDS-R and Women’s Health Questionnaire symptom scores
**Becorpi clinical and biomarker cohort** [43] ** Italy**	Prospective single-arm multidisciplinary cohort N: 20 breast-cancer survivors	Breast-cancer survivors with GSM/VVA 16 hormonal therapy; 3 radiotherapy; 1 chemoradiotherapy; all had surgery	Fractional CO_2_ vaginal laser protocol	No comparator	Pre/post-treatment assessment	Symptoms, VHI, FSFI, FSDS-R, cytokines, vaginal microbiome and adverse effects
**AFLT study for MMK** [44] ** Japan**	Prospective non-randomized single-arm registry-results study N: Target 20; 7 enrolled and reported in registry	Women with breast cancer and vulvovaginal atrophy symptoms Cancer-related low-estrogen state; detailed anticancer therapy not reported	Ablative fractional CO_2_; 3 sessions every 4 weeks	Self-controlled pre/post comparison	One month after treatment; registry also planned 3, 6 and 12 months	VHI, OABSS, vaginal discomfort, urinary symptoms and adverse events

Abbreviations: AI, aromatase inhibitor; BCS, breast cancer survivor; CO_2_, carbon dioxide; FSFI, Female Sexual Function Index; GSM, genitourinary syndrome of menopause; Nd:YAG, neodymium-doped yttrium aluminium garnet; RCT, randomized controlled trial; RF, radiofrequency; VAS, visual analogue scale; VEL, vaginal Er:YAG laser; VHI, Vaginal Health Index. Multiple publications from the same cohort or trial are presented as one study family. For four reports, only a detailed abstract was available to the review team (Jacobsen dose report, Fidecicchi, Quick 12-month report, and Lami). UMIN000027742 is shown separately as registry-results-only evidence and was not treated as a published article. Device-specific terminology and the exact intervention descriptions reported by the study authors are summarized in Supplementary Table S11.

## Data Availability

The data supporting the findings of this study are available within the article and its Supplementary Materials. Further inquiries can be directed to the corresponding author.

## Supplementary Materials

The following supporting information can be downloaded at https://www.mdpi.com/article/10.3390/jcm15197473/s1. Table S1: Complete search strategies for all databases and registries; Table S2: RoB 2 assessment of randomized studies; Table S3: ROBINS-I assessment of the non-randomized comparative study; Table S4: JBI critical appraisal of observational cohort evidence; Table S5: Patient-reported vaginal dryness and dyspareunia outcomes; Table S6: Sexual function, quality of life, and satisfaction; Table S7: Vaginal health, urinary outcomes, and tissue findings; Table S8: Durability and safety; Table S9: GRADE summary of findings; Table S10: Endocrine-therapy representation and subgroup evidence; Table S11: Device-specific and protocol descriptors reported in the included studies. Table S12: Adverse-event ascertainment and reported events by laser modality.
