# Supplementary material for: Vaginal Laser Therapy for Genitourinary Syndrome of Menopause in Breast Cancer Survivors: A Modality-Specific Systematic Review of Efficacy, Durability, and Safety
Source: J Clin Med. 2026 Sep 25;15(19):7473. doi: 10.3390/jcm15197473 (PMC13665872; doi:10.3390/jcm15197473)
Supplement: Supplementary file 1 [file jcm-15-07473-s001.zip › jcm-4588912-supplementary.pdf]

Supplementary Material

Vaginal Laser Therapy for Genitourinary Syndrome of Menopause in Breast Cancer Survivors: A Modality-Specific Systematic Review of Efficacy, Durability, and Safety

Supplementary Tables S1-S12. Reference numbers correspond to the main manuscript.

Supplementary Table S1. Complete search strategies

Review: Vaginal Laser Therapy for Genitourinary Syndrome of Menopause in Breast Cancer Survivors: A Modality-Specific Systematic Review of Efficacy, Durability, and Safety

Information source	Date searched	Complete search strategy	Coverage and limits	Records retrieved
PubMed/MEDLINE	21 August 2026	("Breast Neoplasms"[Mesh] OR "breast cancer"[tiab] OR "breast cancers"[tiab] OR "breast neoplasm*"[tiab] OR "breast carcinoma*"[tiab] OR "breast cancer survivor*"[tiab])<br>AND<br>("Laser Therapy"[Mesh] OR laser*[tiab] OR "CO_2 laser*"[tiab] OR "carbon dioxide laser*"[tiab] OR "Er:YAG"[tiab] OR "erbium laser*"[tiab])	From database inception; no language, publication-year, document-type, or other filters.	85
Scopus	21 August 2026	TITLE-ABS-KEY("breast cancer" OR "breast cancers" OR "breast neoplasm" OR "breast neoplasms" OR "breast carcinoma" OR "breast carcinomas" OR "breast cancer survivor" OR "breast cancer survivors")<br>AND<br>TITLE-ABS-KEY(laser* OR "CO_2 laser" OR "CO_2 lasers" OR "carbon dioxide laser" OR "carbon dioxide lasers" OR "fractional CO_2" OR "fractional carbon dioxide" OR "fractional laser" OR "fractional lasers" OR "microablative laser" OR "microablative lasers" OR "non-ablative laser" OR "nonablative laser" OR "Er:YAG" OR "erbium:YAG" OR "erbium YAG" OR "erbium laser" OR "erbium lasers" OR "vaginal laser" OR "vaginal lasers" OR "genital laser" OR "solid-state vaginal laser")	From database inception; no language, publication-year, document-type, or subject-area limits.	137
Cochrane CENTRAL	22 August 2026	#1 ("breast cancer" OR "breast cancers" OR "breast neoplasm" OR "breast neoplasms" OR "breast carcinoma" OR "breast carcinomas" OR "breast cancer survivor" OR "breast cancer survivors"):ti,ab,kw<br>#2 (laser* OR "CO_2 laser" OR "CO_2 lasers" OR "carbon dioxide laser" OR "carbon dioxide lasers" OR "fractional CO_2" OR "fractional carbon dioxide" OR "fractional laser" OR "fractional lasers" OR "microablative laser" OR "microablative lasers" OR "non-ablative laser" OR "nonablative laser" OR "Er:YAG" OR "erbium:YAG" OR "erbium YAG" OR "erbium laser" OR "erbium lasers" OR "vaginal laser" OR "vaginal lasers" OR "genital laser" OR "solid-state vaginal laser"):ti,ab,kw<br>#3 #1 AND #2	From database inception; no language, year, or study-design/RCT filter.	224
ClinicalTrials.gov	22 August 2026	ClinicalTrials.gov advanced search<br>Condition/disease: Breast Cancer<br>Other terms: ("vaginal laser" OR "CO_2 laser" OR "fractional CO_2" OR "carbon dioxide laser" OR "Er:YAG" OR "erbium laser")<br>Intervention/treatment: left blank<br>Study status: All studies	All recruitment statuses; no location, age, sex, phase, or date limits.	17
WHO ICTRP	22 August 2026	WHO ICTRP Search Portal<br>("breast cancer" OR "breast neoplasm" OR "breast carcinoma") AND (laser OR "vaginal laser" OR "CO_2 laser" OR "carbon dioxide laser" OR "Er:YAG" OR "erbium laser")<br>Recruitment status: All	All recruitment statuses; no country, age, sex, or date limits.	94†

Notes. The search deliberately required only the population and intervention concepts. Terms for genitourinary syndrome of menopause, vulvovaginal atrophy, or individual symptoms were not mandatory, to avoid missing reports that did not use standardized GSM terminology in titles, abstracts, keywords, or registry records. Searches covered each source from inception and used no language restrictions. The terms ‘non-ablative laser’ and ‘nonablative laser’ were retained in the search syntax solely to retrieve reports using those author-supplied descriptors; they were not treated as a separate CO_2 modality in the synthesis.

† The WHO ICTRP export initially contained 96 rows; two non-record header rows were removed, leaving 94 genuine registry records.

Supplementary Table S2. Risk-of-bias assessment of randomized studies using RoB 2

Study and outcome	D1	D2	D3	D4	D5	Overall	Key rationale
LIGHT [6]<br>FSFI at 6 months	Low	Low	Some concerns	Low	Low	Some concerns	Computer-generated block randomization and concealed allocation; participants, treating gynecologist, and relevant assessors were masked. Twelve of 84 randomized participants were excluded from the complete-case analysis (35/42 laser and 37/42 sham), although losses were reasonably balanced. The primary outcome and analysis plan were prespecified in the published protocol and supplement.
Jaber et al. [7]<br>Intercourse dryness and dyspareunia VAS, 1 month after session 3	Some concerns	Low	Some concerns	Some concerns	Some concerns	Some concerns	Randomization used a computer algorithm, but allocation concealment was not described. Participants received a credible sham and were blinded, but blinding success was not tested and the treating physician was unblinded. Two withdrawals occurred in the sham arm and were excluded from analysis. No publicly accessible prespecified protocol or statistical analysis plan was identified in the report.
Gold et al. [5]<br>Patient-reported dryness, dyspareunia, sexual pain, and sexual functioning at 3 months	Some concerns	Some concerns	Some concerns	High	Some concerns	High	An online randomizer was used, but allocation concealment was not reported and one ineligible participant was excluded after randomization. Participants and providers were unblinded; knowledge of treatment could influence subjective symptom and questionnaire reporting. Core NRS denominators were retained, but response counts varied across sexual-health and quality-of-life domains without a complete missing-data account. A trial registration was cited, but a detailed prespecified analysis plan was not available in the report.
Gold et al. [5]<br>Vaginal Health Index at 3 months	Some concerns	Some concerns	Low	High	Some concerns	High	An online randomizer was used, but allocation concealment was not reported; one ineligible participant was excluded after randomization and baseline VHI differed between groups (P=.031). Laser and hyaluronic-acid treatment could not be blinded. VHI includes subjective clinical components, and assessor blinding was not reported. The trial was registered, but a detailed prespecified analysis plan was not available in the report.
LARF/EPMLARF [8,14,15]<br>GSM symptom severity at 10 months	Low	Low	High	High	Some concerns	High	Computer-generated blocked randomization was used and the allocation list was held by the study nurse. The clinical examiner was blinded, but participants were aware of treatment and the principal symptom outcomes were self-reported. Only 58 of 95 enrolled participants completed treatment and 10-month follow-up; the analysis was per protocol. The publication was an interim analysis of an ongoing registered trial, leaving some concern about selective analysis/reporting.
Fidecicchi et al. [17]<br>Superficial dyspareunia VAS at 3 months	Some concerns	Some concerns	Some concerns	High	Some concerns	High	Only a detailed abstract was available. Methods for sequence generation, concealment, blinding, attrition handling, and prespecified analysis were not reported. Both groups received active Er:YAG protocols, but the patient-reported dyspareunia outcome could have been influenced by awareness of protocol differences.

D1, bias arising from the randomization process; D2, bias due to deviations from intended interventions; D3, bias due to missing outcome data; D4, bias in measurement of the outcome; D5, bias in selection of the reported result. Judgments apply to the specified patient-reported or primary efficacy outcome and time point.

Supplementary Table S3. ROBINS-I assessment of the non-randomized comparative intervention study

Okui et al. [16]: VEL+Nd:YAG versus VEL alone; superficial vulvar pain VAS at 24 months

Domain	Judgment	Support for judgment
D1: Confounding	Serious	Treatment was determined by calendar period (VEL from 2016; VEL+Nd:YAG from 2018), creating potential secular confounding. Propensity-score matching included age, menopausal variables, BMI, diabetes, hypertension, and smoking, but did not fully address baseline symptom history, breast-cancer treatment, concomitant care, or other time-varying factors.
D2: Participant selection	Serious	The retrospective cohort required at least two years of follow-up and complete data. Sixteen VEL+Nd:YAG and six VEL participants were excluded after treatment because of shorter follow-up or missing data; selection therefore occurred using postintervention information and differed between groups.
D3: Intervention classification	Low	The two laser strategies were clearly defined and assigned by treatment era; misclassification is unlikely.
D4: Deviations from interventions	Moderate	Protocols were standardized, but the retrospective report did not fully capture concurrent therapies, changes in supportive care, or other deviations across the two calendar periods.
D5: Missing data	Serious	Participants with incomplete two-year data were excluded before matching. Additional FSFI nonresponse occurred at 12 and 24 months. Complete-case analyses may have biased extended-follow-up estimates.
D6: Outcome measurement	Moderate	The main pain outcome was patient-reported VAS. Participants and clinicians were aware of treatment, although the same instruments and follow-up schedule were used in both groups.
D7: Reported-result selection	Moderate	The analysis was planned retrospectively and registration occurred after treatment completion. Multiple outcomes and time points were available without a clearly accessible prespecified analysis plan.
Overall risk of bias	Serious	The study was judged at serious overall risk because serious risk was identified for residual/secular confounding, postintervention selection, and missing extended-follow-up outcome data. No domain was judged critical.

ROBINS-I domains: D1 confounding; D2 selection of participants; D3 classification of interventions; D4 deviations from intended interventions; D5 missing data; D6 outcome measurement; D7 selection of the reported result. Siliquini and other subgroup-comparison cohorts were not treated as non-randomized intervention comparisons and were appraised with the JBI cohort checklist.

Supplementary Table S4. JBI critical appraisal of observational cohort evidence

JBI Critical Appraisal Checklist for Cohort Studies; item-level judgments without an aggregate quality score

Study family	Q1	Q2	Q3	Q4	Q5	Q6	Q7	Q8	Q9	Q10	Q11	Principal limitation
VagLaser dose/histology programme [9,18]	NA	NA	Y	Y	N	NA	Y	Y	Y	N	Y	Small single-centre cohort; no control for confounding and complete-case paired analyses.
Quick/Ohio State cohort [19-21]	NA	NA	Y	Y	N	NA	Y	Y	N	N	Y	Marked attrition at 12 and 24 months; extended-follow-up analyses were complete-case.
Favier 24-month cohort [22]	NA	NA	Y	Y	N	NA	Y	Y	N	N	Y	Retrospective extended-follow-up survivor sample with potential response/selection bias and unadjusted subgroup analyses.
Veron extended-follow-up cohort [23]	NA	NA	Y	Y	N	NA	Y	Y	N	N	Y	Loss to follow-up by the end-of-study assessment and no strategy to address incomplete data.
Salvatore five-session pilot [24,25]	Y	Y	Y	Y	N	NA	Y	Y	Y	NA	Y	Current-versus-previous endocrine-therapy comparison was not adjusted for confounding.
Siliquini BCS/healthy comparison [26]	Y	Y	Y	Y	Y	NA	Y	Y	Y	NA	Y	Age/BMI matching reduced but did not eliminate residual confounding; both groups received laser.
LAAVA pilot [27]	NA	NA	Y	Y	N	NA	Y	Y	Y	NA	Y	Small uncontrolled feasibility cohort; no adjustment for prognostic or treatment-related factors.
Arêas vaginal Er:YAG cohort [28]	NA	NA	Y	Y	N	NA	Y	Y	Y	NA	Y	Small uncontrolled cohort with short post-treatment follow-up.
Gambacciani/Levancini Er:YAG pilot [29,30]	NA	NA	Y	N	N	NA	Y	Y	N	N	Y	Incomplete extended follow-up and limited handling of confounding or missing observations.
Pieralli dyspareunia cohort [31]	NA	NA	Y	Y	N	NA	Y	Y	U	N	Y	Objective vaginal-health assessment was available only for a subset; follow-up completeness was incompletely documented.
Pagano 2016 cohort [32]	NA	NA	Y	Y	N	NA	Y	Y	Y	NA	Y	Retrospective single-arm study without control of confounding.
Pagano 2018 larger cohort [33]	NA	NA	Y	Y	N	NA	Y	Y	Y	N	Y	Retrospective single-arm study; small losses were not addressed analytically.
Mothes dual-phase Er:YAG cohort [34]	NA	NA	Y	U	N	NA	Y	Y	Y	NA	U	Very small retrospective performance cohort; confounder assessment and analytical detail were limited.
Chung Korean cohort [35]	NA	NA	Y	Y	N	NA	Y	Y	N	N	Y	Twenty percent did not complete final assessment; no missing-data strategy.
Ferreira Er:YAG cohort [36]	NA	NA	Y	Y	N	NA	Y	Y	N	N	U	Only 8 of 12 participants had 1-year data; small sample and complete-case analysis.
Perrini dual-wavelength diode cohort [37]	Y	Y	Y	Y	N	NA	Y	Y	Y	NA	Y	Breast-cancer subgroup comparison was not adjusted for baseline differences or treatment history.
Lami CO_2 cohort [38]	Y	Y	Y	Y	N	NA	Y	Y	U	U	U	Detailed abstract only; attrition, confounder handling, and full analytical methods could not be verified.
Lubián-López SSVL cohort [39]	NA	NA	Y	Y	N	NA	Y	Y	N	N	Y	Loss to follow-up through 24 weeks and complete-case analyses.
Hersant EPIONE feasibility cohort [40]	NA	NA	Y	U	N	NA	Y	Y	Y	NA	Y	Small feasibility cohort with limited identification and handling of confounders.
Gittens/Mullen endocrine subgroup [41]	U	Y	Y	N	N	NA	Y	Y	U	U	Y	Breast-cancer subgroup was very small; baseline comparability and follow-up by subgroup were incompletely reported.
Becorpi clinical/biomarker cohort [42]	NA	NA	Y	Y	N	NA	Y	U	Y	NA	Y	Small uncontrolled cohort; timing was adequate for short-term response but not durability.
AFLT registry-results-only study [43]	NA	NA	Y	U	N	NA	U	U	U	U	U	Registry-only results from 7 participants; limited methods, outcome validation, follow-up detail, and analysis reporting.

Judgment key: Y, yes; N, no; U, unclear; NA, not applicable. Q1 and Q2 were marked NA for single-arm cohorts without between-group comparisons; Q6 was marked NA because the studies evaluated change in pre-existing GSM symptoms rather than incident disease.

Q1 groups similar and recruited from the same population; Q2 exposures measured similarly; Q3 exposure measured validly and reliably; Q4 confounders identified; Q5 strategies to address confounding; Q6 participants free of the outcome at baseline; Q7 outcomes measured validly and reliably; Q8 follow-up sufficient; Q9 follow-up complete and losses described; Q10 strategies for incomplete follow-up; Q11 appropriate statistical analysis.

Interpretation: no aggregate JBI score or numerical quality threshold was applied. All studies were retained, and item-level limitations were used when interpreting efficacy, durability, and safety. The most recurrent concerns were absence of strategies to address confounding, incomplete extended follow-up, complete-case analysis, small samples, and limited reporting in abstract- or registry-only evidence.

Supplementary Table S5. Patient-reported vaginal dryness and dyspareunia outcomes

Results are organized by study family and labelled by laser modality to avoid double counting of linked reports. No direct CO_2-versus-Er:YAG comparison was identified; cross-modality contrasts are descriptive only.

Study family	Design / comparison	Vaginal dryness	Dyspareunia	Synthesis note
LIGHT [6]	Sham-controlled RCT; CO_2 laser (n=35) vs sham (n=37); 5 monthly sessions; 6 months	Not reported as a stand-alone symptom outcome.	VAS 0-10, mean ± SD. Laser: 7.3 ± 2.4 to 3.0 ± 2.8; sham: 7.8 ± 2.3 to 3.3 ± 2.5. Between-group difference in change -0.3 (95% CI -1.9 to 1.3), P=.73.	No specific benefit vs sham; different estimand/time point from Jaber.
Jaber [7]	Sham-controlled RCT; CO_2 laser (n=19) vs sham (n=15); 3 sessions; 1 month	VAS 0-10; GEE laser effect ± SE: intercourse dryness -0.03 ± 0.40, P=.944; daily dryness -1.30 ± 0.55, P=.017 (negative favors laser).	VAS 0-10; GEE laser effect ± SE 0.12 ± 0.43, P=.777.	No effect for co-primary symptoms; daily dryness favored laser. Not pooled with LIGHT because estimands/follow-up differed.
Gold [5]	Active-comparator RCT; 43 analyzed after 1 post-randomization exclusion; Er:YAG (n=22) vs hyaluronic acid (n=21); 3 months	Symptom present: 20/22 to 16/22 vs 18/21 to 14/21. Bother NRS median [IQR]: 7 [3-8] to 3 [0-7] vs 4 [0-8] to 2 [0-4]; between-group P=.411.	Present: 19/22 to 15/22 vs 16/21 to 13/21. Bother NRS median [IQR]: 9 [8-10] to 7 [1-9] vs 7 [5-10] to 5 [0-8]; between-group P=.216.	No superiority detected; active comparator and ordinal/median outcomes were not compatible with sham evidence. Nonsignificant P values do not establish equivalence.
LARF/EPMLARF [8,14,15]	Three-arm RCT; CO_2 laser (n=21), radiofrequency (n=19), promestriene (n=18); 10 months	Moderate/severe (VAS >4): CO_2 18/21 to 5/21 at 4 months and 8/21 at 10 months; RF 16/19 to 8/19 and 9/19; promestriene 17/18 to 7/18 and 8/18. Group×time P=.165.	Moderate/severe among evaluable sexually active participants: CO_2 8/12 to 2/12 and 2/12; RF 6/8 to 6/8 and 4/8; promestriene 9/11 to 4/11 and 5/11. Group×time P=.430.	No differential effect; thresholded outcomes and active comparators precluded pooling.
Okui [16]	Non-randomized matched comparison; Er:YAG+Nd:YAG vs Er:YAG (102 per group); 24 months	No direct quantitative symptom outcome.	Superficial vulvar pain VAS mean ± SD: 8.74 ± 0.72 to 4.43 ± 1.38 vs 8.79 ± 0.74 to 8.44 ± 1.09 at 24 months; change -4.30 ± 1.44 vs -0.35 ± 0.93, P<.001.	Comparative but serious risk of confounding; different laser strategies and outcome construct.
Fidecicchi [17]*	Randomized pilot; standard Er:YAG VEL vs hyperstack protocol (n=34 per arm); 3 months	Not reported.	Reduction after the third session: 58% with standard VEL vs 73.5% with hyperstack; greater and persistent improvement with hyperstack, P<.001.	Abstract-only; no arm-level means or variance estimates.
VagLaser dose/histology [9,18]*	Single-arm CO_2 dose programme (n=30; 5 sessions) with linked histology report	Dose report states significant improvement after 3 sessions with further reduction after 5; exact values not available in the supplied abstract.	Reported qualitatively in the dose programme; no extractable arm-level values. Histology report did not provide direct symptom estimates.	No extractable variance or comparator.
Quick/Ohio State [19-21]*	Single-arm CO_2 feasibility cohort; n=59 at 4 weeks, n=39 at 12 months, n=33 at 2 years (paired 2-year VAS analysis n=31)	Moderate/severe: 52/59 (88.1%) at screening to 18/59 (30.5%) at 4 weeks. Individual dryness not separately quantified at 2 years.	Moderate/severe: 48/59 (81.2%) to 18/59 (30.5%) at 4 weeks. Combined Vaginal Assessment Scale change -0.99 (95% CI -1.19 to -0.79), P<.001; paired 4-week-to-2-year change in 31 participants +0.23 (95% CI -0.05 to 0.51), P=.150.	Single arm; combined scale and attrition limit quantitative synthesis.
Favier [22]	Retrospective extended-follow-up CO_2 cohort; breast cancer subgroup n=26; >24 months	NRS mean ± SD at baseline 9.00 ± 1.38; extended-follow-up mean 6.65, P=.004.	Baseline 7.95 ± 2.61; 20.25% reduction at extended follow-up, P=.366; endpoint variance not reported.	Single arm; selected participants retained at extended follow-up and incomplete variance reporting.
Veron [23]	Prospective single-arm CO_2 cohort; n=46; approximately 18 months	No direct quantitative patient-reported dryness estimate.	No direct symptom scale. FSFI pain-domain median [IQR] 1.2 [0-2.0] at baseline, 2.8 [0.9-3.7] at 6 months, and 1.2 [0-2.6] at study end (higher FSFI is better).	Indirect proxy only; not pooled as dyspareunia.
Salvatore [24,25]	Prospective CO_2 pilot; prior endocrine therapy n=25, current therapy n=15; 5 sessions; 20 weeks	VAS mean ± SD: prior therapy 8.6 ± 1.0 to 2.3 ± 1.6; current therapy 9.2 ± 1.0 to 2.7 ± 2.0; both P<.0001.	VAS mean ± SD: prior therapy 8.8 ± 1.3 to 2.0 ± 1.6; current therapy 9.2 ± 1.5 to 2.3 ± 1.2; both P<.0001.	Uncontrolled within-group change; no between-group difference at endpoint.
Siliquini [26]†	Retrospective treated comparison; BCS n=45 vs non-BC controls n=90; 12 months	Narrative means: BCS 8.55 to 3.57 at 3 months and approximately 1.8 at 12 months; controls 7.34 to 1.88 and approximately 1.8.	Narrative means: BCS 8.19 to 5.00 at 3 months and 3.33 at 12 months; controls 8.44 to 2.88 and 2.44.	Both groups received laser; internal table/narrative inconsistency; no untreated effect estimate.
LAAVA [27]	Single-arm CO_2 pilot; n=26; 12 weeks	VAS mean ± SD: 8.31 ± 1.05 at baseline, 5.76 ± 2.28 at 4 weeks, 3.92 ± 2.00 at 8 weeks, 3.21 ± 2.15 at 12 weeks; P<.001.	VAS mean ± SD: 8.83 ± 2.65, 6.23 ± 2.33, 4.83 ± 2.13, and 4.53 ± 3.11; P<.001.	Single-arm repeated measures; change-score SD unavailable.
Arêas [28]	Prospective single-arm Er:YAG cohort; n=24; 1 month after 3 sessions	No direct patient-reported dryness scale; VHI moisture is an objective/clinical proxy and was not treated as symptom dryness.	SPEQ component mean ± SD 3.46 ± 1.66 to 2.23 ± 1.30, P=.012.	Single arm; different outcome scale and no change-score variance.
Gambacciani/Levancini [29,30]	Single-arm Er:YAG pilot; n=37 completers; 18 months	VAS mean ± SD 8.5 ± 1.0 at baseline; 3.8 ± 1.5 at 3 months, 5.5 ± 1.5 at 12 months (both P<.01), and 7.5 ± 1.8 at 18 months (NS vs baseline).	VAS mean ± SD 7.5 ± 1.5; 4.3 ± 1.8 at 3 months, 5.1 ± 1.8 at 12 months (both P<.01), and 6.5 ± 1.8 at 18 months (NS).	Single arm; benefits attenuated by 18 months; change-score SD unavailable.
Pieralli [31]	Prospective single-arm CO_2 cohort; n=50; 30 days after 3 sessions	No direct quantitative symptom outcome.	VAS 0-5 median [range] 5 [1-5] to 3 [1-5], P<.001.	Single arm; median/range reporting.
Pagano 2016 [32]	Retrospective single-arm CO_2 cohort; n=26; 30 days after 3 sessions	VAS 0-10 median [range]: 10 [7-10] at baseline, 6 [0-10] after session 1, 4 [0-7] after session 2, and 2 [0-5] after session 3; P<.0001.	VAS 0-10 median [range]: 9 [5-10], 6 [0-10], 4 [0-9], and 2 [0-5]; P<.0001.	Single arm; possible cohort overlap with Pagano 2018; median/range reporting.

Study family	Design / comparison	Vaginal dryness	Dyspareunia	Synthesis note
Pagano 2018 [33]	Retrospective single-arm CO_2 cohort; n=82 baseline, n=77 post-treatment	Mean 9 to 4; mean reduction 5 (95% CI 4 to 5), P<.001.	Mean 9 to 4; mean reduction 4 (95% CI 3 to 5), P<.001.	Single arm; possible overlap with Pagano 2016; follow-up not suitable for independent pooling.
Mothes [34]	Retrospective single-arm dual-phase Er:YAG cohort; n=16; mean follow-up 8.3 weeks	No direct extractable numerical symptom estimate.	No direct extractable numerical symptom estimate.	VHI and satisfaction reported; no usable primary-outcome effect estimate.
Chung [35]	Prospective single-arm CO_2 cohort; n=24; 12 weeks	VAS mean ± SD 4.7 ± 2.9 at baseline, 3.0 ± 2.1 at 8 weeks, and 2.0 ± 1.8 at 12 weeks; P=.020 for baseline to 8 weeks, P=.060 for baseline to 12 weeks, and P=.060 for 8 to 12 weeks, as reported.	No direct quantitative dyspareunia outcome.	Single arm; the published P values were transcribed as reported despite their apparent discordance with the mean changes.
Ferreira [36]	Prospective single-arm Er:YAG cohort; n=12 baseline, n=10 at 1 month, n=8 at 1 year	Ordinal severity 0-3: mean 2.50 at baseline, 0.25 at 1 month, and 0.125 at 1 year.	Ordinal severity 0-3: mean 1.85, 0.714, and 0.428. Overall symptom-severity P=.0078; item-specific P values not reported.	Small single arm with attrition and ordinal outcomes.
Perrini [37]	Prospective treated cohort; breast cancer subgroup n=13; 4 weeks after 3 sessions	No direct quantitative symptom outcome.	VAS mean ± SD 7.77 ± 1.79 at baseline, 6.46 ± 1.94 before session 2, 4.62 ± 2.26 before session 3, and 4.00 ± 2.48 at follow-up; P<.001.	Small subgroup; all participants treated; no change-score variance.
Lami [38]*	Single-arm CO_2 cohort (author-described as non-ablative); n=26; 1 month after 3 sessions	Reported as significantly improved; exact numerical estimates unavailable in the supplied abstract.	No direct symptom estimate; FSFI pain domain reportedly improved.	Abstract-only; no extractable primary-outcome variance.
Lubián-López [39]	Prospective single-arm solid-state vaginal laser; n=27 at 10 weeks, n=24 at 24 weeks	No direct quantitative symptom outcome.	VAS mean ± SD 7.5 ± 1.6 at baseline, 3.4 ± 2.4 at 10 weeks, and 2.4 ± 2.4 at 24 weeks; P<.001.	Single arm; change-score variance not fully reported.
Hersant [40]	Prospective single-arm CO_2 feasibility cohort; n=20; 6 months	No direct extractable numerical symptom estimate.	No direct extractable numerical symptom estimate.	VHI and sexual-distress outcomes only for the present review endpoints.
Gittens/Mullen [41]	Retrospective treated cohort; endocrine-treated BCS subgroup n=8; after 3 sessions	Wong-Baker Faces scale improvement from baseline: 3.25 ± 3.33 points, P=.028.	Wong-Baker Faces scale improvement: 4.25 ± 3.45 points, P=.010.	Very small treated subgroup; endpoint means not reported.
Becorpi [42]	Prospective single-arm CO_2 cohort; n=20; after 3 sessions	Ordinal symptom median [IQR] 2 [2-3] to 2 [1-2], P=.002. Objective mucosal dryness 2 [2-2.75] to 1 [0-1.75], P<.001.	Ordinal symptom median [IQR] 2 [2-3] to 2 [1-2], P=.006.	Single arm; ordinal data and no change-score variance.
AFLT registry [43]	Registry-results-only single-arm CO_2 study	No extractable numerical result.	No extractable numerical result.	Registry evidence only; insufficient results for synthesis.

*Abbreviations: BCS, breast cancer survivor; CI, confidence interval; CO2, carbon dioxide; GEE, generalized estimating equations; IQR, interquartile range; Nd:YAG, neodymium-doped yttrium aluminium garnet; NRS, numerical rating scale; NS, not significant; RCT, randomized controlled trial; RF, radiofrequency; SD, standard deviation; SE, standard error; SPEQ, Short Personal Experiences Questionnaire; VAS, visual analogue scale; VEL, vaginal Er:YAG laser; VHI, Vaginal Health Index. Lower symptom scores indicate improvement unless stated otherwise. *At least one linked report was available only as a detailed abstract. †The Siliquini article contains an internal inconsistency between the narrative and Table 2 at 12 months; narrative values are shown. No pair of studies met the prespecified clinical and statistical compatibility criteria for a confirmatory meta-analysis of vaginal dryness or dyspareunia.* The outcome-level compatibility assessment for the two sham-controlled CO_2 trials is presented below.

Outcome-level compatibility assessment for the two sham-controlled CO_2 trials (Supplementary Table S5)

Primary outcome	LIGHT [6]	Jaber [7]	Clinical and time-point incompatibility	Estimand and variance incompatibility	Pooling decision
**Vaginal dryness**	No stand-alone vaginal-dryness outcome corresponding to the Jaber constructs was reported.	Daily dryness and intercourse dryness were analyzed as separate outcomes using GEE; assessment was 1 month after session 3.	LIGHT used five monthly sessions with assessment at 6 months; Jaber used three sessions every 3-4 weeks with assessment 1 month after session 3. The dryness constructs were also not identical.	No LIGHT estimate existed for a matching dryness construct; therefore a common effect estimate and corresponding variance could not be formed.	Not poolable: no common primary-outcome construct was reported by both trials.
**Dyspareunia**	VAS 0-10; mean change by arm and between-group difference in change at 6 months after five monthly sessions: -0.3 (95% CI -1.9 to 1.3).	VAS 0-10; GEE treatment coefficient 1 month after session 3: 0.12 (SE 0.43).	Five monthly sessions with a 6-month assessment versus three sessions every 3-4 weeks with assessment 1 month after session 3.	Between-group difference in mean change versus a GEE model coefficient. Jaber reported a model-based SE but not arm-level change means and variances compatible with the LIGHT analysis.	Not pooled: the common scale did not overcome the non-equivalent treatment exposure, time point, and estimand.

Compatibility was assessed at the outcome level. The presence of two sham-controlled studies was not considered sufficient for pooling when intervention exposure, outcome construct, assessment time point, and reported estimand were not aligned.

Supplementary Table S6. Sexual function, quality of life, and satisfaction

Comparative results are prioritized and modality-labelled. Cohort results describe response patterns and durability, not comparative efficacy; cross-modality contrasts are descriptive only.

Study	Modality / design	Outcome and follow-up	Key result	Interpretation
LIGHT [6]	CO_2 — Sham-controlled RCT	FSFI; body image; SF-12; 6 mo	FSFI improved in both groups, but change did not differ: laser 5.2 vs sham 7.9; between-group difference 2.8 (95% CI -1.0 to 6.5), P=.15. Body image and SF-12 also showed no between-group differences.	No laser-specific benefit
Jaber [7]	CO_2 — Sham-controlled RCT	FSFI; 1 mo after 3 sessions; open phase after 6	No significant time or laser effect on total or domain FSFI in the comparative phase. After 6 open-label sessions, total FSFI change was 4.33 ± 2.45, P=.078; satisfaction domain improved, but the total change was neither statistically nor clinically significant.	No comparative benefit
Gold [5]	Er:YAG — Laser vs HA RCT	EORTC QLQ-BR45, SHQ-C22, pelvic-floor questionnaire, satisfaction; 3 mo	At 3 months, EORTC SHQ-C22 sexual pain was 33.3 (IQR 25.0-50.0) with Er:YAG versus 54.2 (8.3-66.7) with HA (P=.390), and sexual satisfaction was 28.13 (15.6-39.3) versus 31.7 (23.4-45.3) (P=.388). EORTC QLQ-BR45 sexual functioning was 33.3 (0-50.0) versus 33.3 (16.7-50.0) (P=.677); no QLQ-BR45 domain showed a detected between-group difference.	No superiority detected; small unblinded trial with variable questionnaire response counts and no contrast estimates or confidence intervals
LARF/EPMLARF [8]	CO_2 — CO_2 vs RF vs promestriene RCT	FSFI; I-QOL; satisfaction; 4 and 10 mo	FSFI improved over time in all groups (time P<.001), without group-by-time interaction (P=.271). At 10 mo, median FSFI was 25.6, 25.0, and 23.5, respectively. I-QOL interaction P=.182. Satisfaction was high in all groups.	No differential sexual-function effect
Okui [16]	Er:YAG+Nd:YAG vs Er:YAG — Non-randomized two-strategy cohort	FSFI; 12 and 24 mo	After propensity matching, total FSFI favored Er:YAG+Nd:YAG at 12 mo (20.89 vs 18.06, P=.001) and 24 mo (20.47 vs 15.88, P<.001).	Serious ROBINS-I risk; not causal
VagLaser [9]	CO_2 — Single-arm dose cohort	FSFI, SCS-W; after sessions 3-5	Abstract reports increasing FSFI and decreasing sexual-concern scores, with additional improvement after later sessions.	Abstract-only; no control
Quick/Ohio State [19-21]	CO_2 — Single-arm prospective cohort	FSFI, FSDS-R; 4 wk, 12 and 24 mo	At 4 wk, mean FSFI change was 9.67 (95% CI 7.27 to 12.08). Improvement persisted at 2 y; FSFI change from year 1 to 2 was -0.83 (95% CI -3.07 to 2.38), P=.794, and FSDS-R change was -2.85, P=.227.	Persistent within-group signal; high attrition
Favier [22]	CO_2 — Retrospective extended-follow-up cohort	FSFI, SF-12, satisfaction; >24 mo	No significant change in FSFI or SF-12 during extended follow-up. Overall, 41% were satisfied, 21% neutral, and 38% dissatisfied; satisfaction was 42% in the breast-cancer subgroup.	Mixed durability; no control
Veron [23]	CO_2 — Prospective single-arm cohort	FSFI, SF-12; median 19 mo	FSFI increased at 6 mo (mean change 8.3, P<.0001) and remained above baseline at study end (mean change 4.3, P=.01), but pain and desire domains returned to baseline. SF-12 did not change.	Attenuated within-group improvement during extended follow-up
Salvatore [24,25]	CO_2 — Prospective single-arm cohort	FSFI, SF-12, satisfaction; 20 wk	Among sexually active women, FSFI increased from 19.3 to 26.8 (P<.001); SF-12 components improved. At 20 wk, 77.5% were satisfied or very satisfied.	Short-term uncontrolled improvement
LAAVA [27]	CO_2 — Prospective single-arm pilot	FSFI; patient-reported sexual function/QoL; 12 wk	FSFI and all six domains improved (P<=.036). Fifty percent reported improved sexual function and 65% improved quality of life.	Short-term uncontrolled improvement
Arêas [28]	Er:YAG — Prospective single-arm cohort	SPEQ; 1 mo after session 3	Odds of being above the prespecified sexual-function threshold improved (OR 8.0; P=.04); arousal, enjoyment, orgasm, partner satisfaction, and dyspareunia also improved.	Short-term uncontrolled improvement
Pieralli [31]	CO_2 — Prospective single-arm cohort	Satisfaction; mean telephone follow-up 11 mo	High patient-reported satisfaction was reported after treatment; validated multidomain sexual-function data were not reported.	Uncontrolled; limited outcome detail
Pagano [32,33]	CO_2 — Retrospective single-arm cohorts	Sexual/genitourinary symptom VAS; 1 mo	Both cohorts reported short-term reductions in sexual and genitourinary symptom severity; validated multidomain sexual-function outcomes were not available.	Uncontrolled and short term
Mothes [34]	Er:YAG — Retrospective single-arm cohort	Subjective satisfaction; mean 8.3 wk	Most participants reported subjective improvement/satisfaction after a single dual-phase Er:YAG treatment.	Selected postoperative population
Chung [35]	CO_2 — Prospective single-arm cohort	Satisfaction; 12 wk	High satisfaction accompanied short-term symptom and VHI improvement; no untreated comparison.	Short-term uncontrolled evidence

Study	Modality / design	Outcome and follow-up	Key result	Interpretation
Ferreira [36]	Er:YAG — Prospective single-arm pilot	FSFI, satisfaction; 1 mo and 1 y	FSFI and satisfaction improved after treatment; only eight participants remained at 1 y.	Very small cohort with attrition
Lami [38]	CO_2 (author-described as non-ablative) — Prospective single-arm cohort	FSFI, FSDS, MENQOL; 1 mo	Abstract reports improvement in total FSFI and selected lubrication, orgasm, and pain domains and the MENQOL sexual domain; FSDS and several FSFI domains did not improve.	Detailed abstract only
Lubián-López [39]	other solid-state — Prospective single-arm cohort	FSFI, EORTC QLQ-BR23, satisfaction; 10 and 24 wk	FSFI was unchanged at 10 wk (18.8 to 21.05, P=.13) but improved at 24 wk (23.7; P<.001). Satisfaction averaged 4.7/5; 95.7% were at least satisfied.	Delayed within-group improvement
Hersant [40]	CO_2 — Prospective feasibility cohort	Sexual distress; satisfaction; 6 mo	Sexual distress and satisfaction improved during follow-up; mean satisfaction was 7.15/10 at 6 mo.	Small uncontrolled feasibility study
Gittens/Mullen [41]	CO_2 — Retrospective subgroup cohort	FSFI, FSDS-R, WHQ; through session 3	Breast-cancer endocrine-therapy subgroup showed within-group improvement in sexual-function and symptom scores.	Subgroup n=8; indirect and imprecise
Becorpi [42]	CO_2 — Prospective single-arm cohort	FSFI, FSDS-R; post-treatment	Reported improvement in sexual function and sexual distress after treatment, alongside clinical and biomarker changes.	Small uncontrolled cohort

Supplementary Table S7. Vaginal health, urinary outcomes, and tissue findings

VHI is partly clinician rated. Results are modality-labelled; histological and biomarker findings are mechanistic and should not be equated with symptom benefit or comparative modality superiority.

Study	Modality / design	Outcome and follow-up	Key result	Interpretation
LIGHT [6]	CO_2 — Sham-controlled RCT	VHI, pH, VMI, epithelial thickness, elasticity; 6 mo	VHI change did not differ (laser 3.3 vs sham 5.0; between-group difference 1.4, 95% CI -0.6 to 3.5; P=.17). No between-group differences in pH, VMI, thickness, or elasticity.	No laser-specific objective benefit
Jaber [7]	CO_2 — Sham-controlled RCT	VHI, modified Schirmer hydration, pH; 1 mo	Laser coefficients favored treatment for VHI (2.26 ± 0.50; P<.001), hydration (3.24 ± 1.13; P=.004), and pH (-0.31 ± 0.14; P=.021), but several individual symptoms favored sham.	Mixed comparative profile
Gold [5]	Er:YAG — Laser vs HA RCT	VHI primary; pelvic-floor/urinary symptoms; 3 mo	Baseline VHI differed between groups: median 9 (IQR 8-13) with Er:YAG versus 13 (10-16) with HA (P=.031). Both groups improved (P=.001), but no between-group difference was detected at 3 months (P=.232). Follow-up arm values, a contrast estimate, and its confidence interval were not reported.	No superiority detected; baseline imbalance and incomplete effect reporting
LARF/EPMLARF [8,14,15]	CO_2 — CO_2 vs RF vs promestriene RCT	VHI, urinary symptoms, I-QOL; vaginal/vulvar histology; 4 and 10 mo	VHI improved over time without interaction (P=.320). Urinary-symptom frequency interaction favored energy groups (P=.040), but I-QOL interaction was not significant (P=.182). No post-treatment histological differences or tissue damage were identified.	Comparator-consistent clinical response; no differential histology
Okui [16]	Er:YAG+Nd:YAG vs Er:YAG — Non-randomized two-strategy cohort	VHI; 12 and 24 mo	VHI improved similarly at 12 mo (16.21 vs 16.59) and regressed by 24 mo (14.92 in both groups); no between-group difference.	Vaginal-health benefit waned
VagLaser [9,18]	CO_2 — Single-arm dose/histology cohort	VHI and urinary scales after sessions 3-5; paired biopsy after session 5	Abstract reports progressive VHI and selected urinary/sexual improvements. Elastin increased 33.1% to 56.8% (P<.01) and collagen III 44.5% to 58.4% (P=.03); epithelial morphology, total collagen, and collagen I did not change.	Preliminary stromal remodeling; no control
Quick/Ohio State [19-21]	CO_2 — Single-arm prospective cohort	UDI-6, pH and physical findings; 4 wk to 2 y	UDI-6 improved at 4 wk (mean change -8.85, 95% CI -12.45 to -4.75) but approached baseline by 2 y. Vaginal pH and clinical findings improved short term.	Urinary durability uncertain
Veron [23]	CO_2 — Prospective single-arm cohort	pH, cytology, Ditrovie urinary QoL; median 19 mo	Small pH improvement at 6 mo was not robust after sensitivity analysis; cytologic maturation did not change. Ditrovie improved at 6 mo but was not different from baseline at study end.	Objective/urinary benefits not sustained
Salvatore [24,25]	CO_2 — Prospective single-arm cohort	VHI, SF-12, dysuria; 20 wk	VHI increased from 10.8 to 21.9 (P<.001); dysuria and other symptoms improved. Similar patterns were reported by current versus previous endocrine-therapy status.	Large short-term within-group change
Siliquini [26]	CO_2 — BCS vs non-BC treated cohort	VHI, VVHI; through 12 mo	VHI and VVHI improved after laser in both treated groups; breast-cancer survivors started with worse values and improved more slowly.	No untreated efficacy control
Arêas [28]	Er:YAG — Prospective single-arm cohort	VHI and components; through session 3	VHI increased from 11.88 at baseline to 15.63 after session 1 and 17.38 after session 2 (P<.001); pH did not significantly change.	Short-term clinician-rated change
Gambacciani/Levancini [29,30]	Er:YAG — Prospective single-arm cohort	VHI; 1-18 mo	VHI increased from 8.1 to 21.0 after session 3 and remained improved through 12 mo, but was 14.8 at 18 mo and no longer significantly different from baseline.	Clear attenuation by 18 mo
Pieralli [31]	CO_2 — Prospective single-arm cohort	VHI in first 36 participants; short term	VHI increased after three CO_2 sessions; the study did not include a control and VHI was unavailable for the full cohort.	Partial objective-outcome sample
Mothes [34]	Er:YAG — Retrospective single-arm cohort	VHI and pH; mean 8.3 wk	VHI and pH improved after one dual-phase Er:YAG treatment in a selected postoperative urogynecologic cohort.	Indirect selected population
Chung [35]	CO_2 — Prospective single-arm cohort	VHI; 12 wk	VHI and symptom scores improved after three sessions.	Short-term uncontrolled evidence
Ferreira [36]	Er:YAG — Prospective single-arm pilot	VHI, cytology/biopsy; 1 mo and 1 y	Clinical and tissue measures suggested improvement after treatment, but the cohort was very small and follow-up incomplete.	Exploratory tissue evidence
Perrini [37]	dual-wavelength diode — Single-arm subgroup cohort	VHI, VMI; 4 wk	VHI and VMI improved after dual-wavelength diode treatment, including the breast-cancer subgroup.	Subgroup without untreated control
Lami [38]	CO_2 (author-described as non-ablative) — Prospective single-arm cohort	VHI, VuHI; 1 mo	Abstract reports significant VHI/VuHI and symptom improvement, irrespective of aromatase inhibitor, tamoxifen, or no adjuvant therapy.	Detailed abstract only
Lubián-López [39]	other solid-state — Prospective single-arm cohort	VHI, VuHI, pH, VMI; 10 and 24 wk	VHI increased 10.6 to 17.1 at 10 wk and remained above baseline at 24 wk (14.6; P<.001); VuHI, pH, and VMI also improved.	Within-group response; some attenuation
Hersant [40]	CO_2 — Prospective feasibility cohort	VHI components and pH; 6 mo	VHI components improved through 6 mo; vaginal pH decreased only slightly.	Small uncontrolled cohort
Becorpi [42]	CO_2 — Prospective single-arm cohort	VHI, symptoms, cytokines, microbiome; post-treatment	Clinical vaginal-health and symptom improvements were accompanied by exploratory inflammatory and microbiome changes.	Hypothesis-generating biomarkers
AFLT [43]	CO_2 — Registry-results-only cohort	VHI, OABSS, discomfort and urinary outcomes	Registry summary reports pre/post assessments in seven participants, but outcome detail and variance were insufficient for synthesis.	Unpublished, highly imprecise

Supplementary Table S8. Durability and safety

Durability was operationally defined for this review as persistence of a reported outcome at ≥6 months, and follow-up at ≥12 months was categorized as extended follow-up. These thresholds organize the evidence and do not imply established long-term treatment efficacy or safety. Controlled evidence extended only to approximately 10 months; all ≥12-month data came from uncontrolled cohorts with substantial attrition or selective retention. Results are modality-labelled. Absence of a reported serious event does not demonstrate safety when cohorts are small, follow-up is incomplete, or adverse-event surveillance is not prespecified.

Study / modality	Longest follow-up	Durability finding	Safety and tolerability finding
LIGHT [6] — CO_2	6 mo	No laser-specific efficacy signal; both groups improved with co-interventions.	No severe related event. Mild/moderate related events occurred in both groups. Estradiol did not increase in the laser arm.
Jaber [7] — CO_2	1 mo after session 3; open phase after session 6	Comparative symptom effects were mixed; additional open-label sessions produced further within-group changes.	Spotting/discomfort: 5/19 laser vs 7/15 sham, P=.288. One cystitis requiring antibiotics in each group; no serious event reported.
Gold [5] — Er:YAG	3 mo	Both groups improved, but the trial did not detect superiority of Er:YAG over HA; follow-up ended at 3 months.	The authors reported no adverse effects. With 43 analyzed participants and 3 months of follow-up, uncommon or delayed harms cannot be excluded.
LARF/EPMLARF [8,14,15] — CO_2	10 mo	Clinical improvements generally persisted but did not differ among CO_2, RF, and promestriene; urinary pattern favored energy groups.	Pain and mild bleeding occurred; some withdrawals due to procedural pain. No burns, serious complication, or clinical/histological tissue damage.
Okui [16] — Er:YAG+Nd:YAG vs Er:YAG	24 mo	Combined Er:YAG+Nd:YAG pain and FSFI signals persisted; VHI regressed in both groups and Er:YAG-alone pain recurred.	Transient heat, mild pain, discharge, edema, dryness, itching, and one transient de novo incontinence event; no event persisted beyond 1 y.
Fidecicchi [17]	3 mo	Hyperstack protocol produced greater short-term dyspareunia reduction than standard VEL.	Safety outcomes were not sufficiently detailed in the supplied abstract.
VagLaser [9,18] — CO_2	After sessions 3-5	Dose report suggested incremental improvement through five sessions; tissue follow-up was 4-6 wk after session 5.	Dose report stated treatment was well tolerated with no serious adverse event; histology report did not identify epithelial injury.
Quick/Ohio State [19-21] — CO_2	24 mo	Combined symptom and sexual-function improvements persisted among retained participants; UDI moved toward baseline.	Grade 1-2 discharge and dryness were reported initially; no grade >=3 event at initial or 2-y follow-up.
Favier [22] — CO_2	>=24 mo; mean about 3.5 y	Selected symptom improvements persisted, but FSFI/SF-12 did not and 38% were dissatisfied.	No serious treatment-related event was reported during short or extended follow-up. One atypical endometrial hyperplasia in an HRT user at 3 y; causal relation not established.
Veron [23] — CO_2	Median 19 mo	Sexual-function benefit attenuated; urinary QoL returned toward baseline; 58% requested maintenance.	No major adverse event reported; extended-follow-up ascertainment relied on cohort retention.
Salvatore [24,25] — CO_2	20 wk	Short-term symptom, VHI, FSFI, and QoL improvement.	No infection, worsening symptom, or other treatment-related adverse event recorded.
Siliquini [26] — CO_2	12 mo	VHI/VVHI and symptoms remained improved, with slower response in breast-cancer survivors.	No adverse event reported in either treated group.
LAAVA [27] — CO_2	12 wk	Short-term symptom and sexual-function improvement.	No serious adverse event reported; small feasibility sample.
Arêas [28] — Er:YAG	1 mo after session 3	Short-term VHI and sexual-function improvement.	One candidiasis and one acute cystitis; both resolved with treatment.
Gambacciani/Levancini [29,30] — Er:YAG	18 mo	Dryness, dyspareunia, and VHI effects waned and were no longer significant at 18 mo.	No adverse event reported in the available report; uncontrolled follow-up.
Pieralli [31] — CO_2	Mean 11 mo	Dyspareunia and satisfaction improvement was reported; objective assessment was incomplete.	No major complication reported; safety assessment was not standardized.
Pagano [32] — CO_2	1 mo	Short-term symptom reduction.	Procedural discomfort and transient reactions were monitored; no serious event reported.
Pagano [33] — CO_2	1 mo	Short-term sexual and genitourinary symptom reduction.	Five participants did not complete all cycles; no serious event reported.
Mothes [34] — Er:YAG	Mean 8.3 wk	Short-term VHI/pH and satisfaction improvement after one session.	No adverse complication reported in the selected postoperative cohort.
Chung [35] — CO_2	12 wk	Short-term symptom, VHI, and satisfaction improvement.	No serious adverse event reported; limited follow-up.
Ferreira [36] — Er:YAG	1 y	Clinical/tissue response was reported, but only eight participants remained at 1 y.	No serious event reported; sample too small for uncommon harms.
Perrini [37] — dual-wavelength diode	4 wk	Short-term VHI/VMI and dyspareunia improvement.	No significant adverse event reported; procedural pain was assessed.
Lami [38] — CO_2 (author-described as non-ablative)	1 mo	Short-term VHI/VuHI, symptom, and selected sexual-function improvement.	No adverse event reported in the abstract; full methods for surveillance unavailable.
Lubián-López [39] — other solid-state	24 wk	Most clinical measures remained improved, with some attenuation by 24 wk.	No relevant side effect reported; satisfaction high.
Hersant [40] — CO_2	6 mo	VHI and sexual-distress improvement through follow-up.	Mean procedural pain 4.7/10; two moderate bleeding events within 24 h; no other adverse effect reported.
Gittens/Mullen [41] — CO_2	Through session 3	Within-group symptom/sexual-function improvement in subgroup n=8.	No adverse event documented; retrospective subgroup evidence.
Becorpi [42] — CO_2	Post-treatment	Clinical and exploratory biomarker improvement.	No serious adverse effect reported; small cohort.
AFLT [43] — CO_2	Registry planned to 12 mo	Seven participants reported; durability data were insufficient.	Registry summary provided insufficient event detail to evaluate uncommon or delayed harms.

Supplementary Table S9. GRADE summary of findings

GRADE ratings were defined for specific intervention-comparator-outcome questions. Sham-controlled CO_2 rows address laser-specific efficacy. Er:YAG-versus-hyaluronic-acid rows address comparative effectiveness against an active nonhormonal treatment and do not isolate a laser-specific effect. Observational evidence was considered supportive for describing durability and harms but did not raise certainty because of uncontrolled designs and serious residual bias. For single-study contrasts, inconsistency was recorded as not assessable and was not itself a reason for downgrading. For Gold et al., published arm-level medians, interquartile ranges, and P values were used; no between-group effect estimate with a 95% confidence interval was available. Nonsignificant P values were not interpreted as evidence of equivalence.

Outcome	Evidence base	Comparative finding	Risk of bias	Other GRADE domains	Certainty
Vaginal dryness (CO_2 vs sham)	2 CO_2 sham-controlled RCTs; n=106 analyzed in comparative phases [6,7]	LIGHT did not show a specific laser effect; Jaber found no effect for intercourse dryness but improvement in daily dryness.	Serious/some concerns	Downgrade for inconsistency and imprecision; differing definitions and schedules.	Very low (⊕◯◯◯)
Dyspareunia (CO_2 vs sham)	2 CO_2 sham-controlled RCTs; n=106 analyzed [6,7]	Neither trial showed a laser-specific reduction after 3-5 sessions.	Some concerns	Downgrade for imprecision; small samples and confidence intervals compatible with benefit or harm.	Low (⊕⊕◯◯)
Overall sexual function (FSFI) (CO_2 vs sham)	2 CO_2 sham-controlled RCTs; n=106 analyzed [6,7]	No laser-specific improvement in either trial.	Some concerns	Downgrade for imprecision; schedules/follow-up differed but direction was consistent.	Low (⊕⊕◯◯)
Vaginal Health Index (CO_2 vs sham)	2 CO_2 sham-controlled RCTs; n=106 analyzed [6,7]	LIGHT showed no benefit; Jaber reported a laser-associated increase.	Some concerns	Downgrade for inconsistency and imprecision; clinician-rated outcome and different schedules.	Very low (⊕◯◯◯)
Vaginal dryness bother (Er:YAG vs hyaluronic acid)	1 active-comparator RCT; n=43 analyzed after 1 post-randomization exclusion (Er:YAG n=22; hyaluronic acid n=21), 3 months [5]	NRS median (IQR): Er:YAG 7 (3-8) to 3 (0-7); hyaluronic acid 4 (0-8) to 2 (0-4). No between-group difference was detected (P=.411).	Serious (high outcome-specific RoB 2: unblinded self-report; allocation concealment unclear; post-randomization exclusion)	No indirectness for this active-comparator question; very serious imprecision (single small trial; no between-group effect estimate or 95% CI; clinically important differences could not be excluded); inconsistency not assessable and not downgraded.	Very low (⊕◯◯◯)
Dyspareunia bother (Er:YAG vs hyaluronic acid)	1 active-comparator RCT; n=43 analyzed after 1 post-randomization exclusion (Er:YAG n=22; hyaluronic acid n=21), 3 months [5]	NRS median (IQR): Er:YAG 9 (8-10) to 7 (1-9); hyaluronic acid 7 (5-10) to 5 (0-8). No between-group difference was detected (P=.216).	Serious (high outcome-specific RoB 2: unblinded self-report; allocation concealment unclear; post-randomization exclusion)	No indirectness for this active-comparator question; very serious imprecision (single small trial; no between-group effect estimate or 95% CI; clinically important differences could not be excluded); inconsistency not assessable and not downgraded.	Very low (⊕◯◯◯)
Sexual pain (EORTC SHQ-C22; Er:YAG vs hyaluronic acid)	1 active-comparator RCT; up to n=43, with domain response counts varying; 3 months [5]	Median (IQR) at 3 months: Er:YAG 33.3 (25.0-50.0); hyaluronic acid 54.2 (8.3-66.7). No between-group difference was detected (P=.390).	Serious (high outcome-specific RoB 2: unblinded self-report; variable response counts; selective-result concerns)	No indirectness for this active-comparator question; very serious imprecision (single small trial; no between-group effect estimate or 95% CI; clinically important differences could not be excluded); inconsistency not assessable and not downgraded.	Very low (⊕◯◯◯)
Sexual functioning (EORTC QLQ-BR45; Er:YAG vs hyaluronic acid)	1 active-comparator RCT; up to n=43, with domain response counts varying; 3 months [5]	Median (IQR) at 3 months: Er:YAG 33.3 (0-50.0); hyaluronic acid 33.3 (16.7-50.0). No between-group difference was detected (P=.677).	Serious (high outcome-specific RoB 2: unblinded self-report; variable response counts; selective-result concerns)	No indirectness for this active-comparator question; very serious imprecision (single small trial; no between-group effect estimate or 95% CI; clinically important differences could not be excluded); inconsistency not assessable and not downgraded.	Very low (⊕◯◯◯)
Vaginal Health Index (Er:YAG vs hyaluronic acid)	1 active-comparator RCT; n=43 analyzed after 1 post-randomization exclusion (Er:YAG n=22; hyaluronic acid n=21), 3 months [5]	Baseline medians differed: Er:YAG 9 (IQR 8-13) vs hyaluronic acid 13 (10-16; P=.031). Both groups improved (P=.001); no between-group difference was detected at 3 months (P=.232). Follow-up arm values were not reported.	Serious (high outcome-specific RoB 2: assessor masking unreported; subjective VHI components; baseline imbalance; post-randomization exclusion)	No indirectness for this active-comparator question; very serious imprecision (single small trial; no between-group effect estimate or 95% CI; clinically important differences could not be excluded); inconsistency not assessable and not downgraded.	Very low (⊕◯◯◯)
Serious adverse events	5 randomized studies (3 CO_2 and 2 Er:YAG) plus observational cohorts [5-8,17]	No serious treatment-related event was reported, but events were uncommon and surveillance varied.	Serious limitations	Major downgrade for very serious imprecision and indirect/variable ascertainment of rare and delayed harms.	Very low (⊕◯◯◯)

Supplementary Table S10. Endocrine-therapy representation and subgroup evidence

This table summarizes the evidence available for clinically relevant endocrine-therapy strata. It describes representation and subgroup reporting; it does not establish effect modification by endocrine regimen.

Study / family	Endocrine-therapy representation	Laser evidence and follow-up	Treatment-specific inference and limitation
LIGHT [6]	All participants were receiving aromatase inhibitors.	CO_2 versus sham RCT; 6 months.	Direct comparative evidence in aromatase-inhibitor users, but no comparison with tamoxifen, ovarian suppression, or no current endocrine therapy.
LARF/EPMLARF [8,14,15]	All participants were receiving adjuvant endocrine therapy; tamoxifen and aromatase inhibitors were represented.	CO_2 versus radiofrequency versus promestriene randomized trial; 10 months.	Outcomes were not reported as regimen-specific effect estimates; differential response by endocrine therapy cannot be inferred.
Salvatore [24,25]	Previous endocrine therapy n=25; current endocrine therapy n=15.	CO_2 single-arm cohort with internal current-versus-previous therapy comparison; 20 weeks.	Dryness and dyspareunia improved in both groups, with no between-group difference at the endpoint; comparison was non-randomized and unadjusted.
Veron [23]	36/46 receiving endocrine therapy: tamoxifen n=6, aromatase inhibitor n=24, aromatase inhibitor plus LHRH agonist n=6.	CO_2 single-arm cohort; median follow-up 19 months.	Provides the only explicit ovarian-suppression subgroup count, but no separate regimen-specific outcome estimates were reported.
Chung [35]	Among 24 completers: tamoxifen n=11, aromatase inhibitor n=1, no endocrine therapy n=12.	CO_2 single-arm cohort; 12 weeks.	Regimen distribution was reported, but the study did not provide a reliable treatment-stratified effect estimate.
Lami [38]	Aromatase-inhibitor, tamoxifen, and no-adjuvant-therapy subgroups.	CO_2 single-arm cohort reported in a detailed abstract; 1 month.	Improvement was reported irrespective of endocrine-therapy status, but arm-level estimates and variances were unavailable.
Gittens/Mullen [41]	Endocrine-treated breast-cancer subgroup n=8.	CO_2 retrospective treated subgroup; through the third session.	Within-group sexual-function and symptom improvement only; very small subgroup and no comparison across endocrine regimens.
Other mixed-exposure studies [5,7,9,16,19-22,26-28,31-33,39,42]	Current, previous, or unspecified aromatase-inhibitor and/or tamoxifen exposure was reported in varying detail.	Active-comparator or observational studies with heterogeneous protocols and follow-up.	Endocrine therapy was described, but outcome reporting was insufficient for a reliable regimen-specific estimate or effect-modification analysis.

Abbreviations: AI, aromatase inhibitor; CO_2, carbon dioxide; ET, endocrine therapy; LHRH, luteinizing-hormone-releasing hormone; RCT, randomized controlled trial. No study randomized participants by endocrine regimen or was adequately powered to compare response among aromatase inhibitors, tamoxifen, ovarian suppression, and no current endocrine therapy.

Supplementary Table S11. Device-specific and protocol descriptors reported in the included studies

This table preserves the device and protocol terminology reported by the original study authors. These descriptors were not used to infer comparative efficacy between CO_2 and Er:YAG modalities.

Study / family	Device-specific and protocol description as reported
LIGHT [6] Spain	Fractional CO_2 laser, 5 monthly sessions
Jaber sham-controlled RCT [7] Israel	Fractional CO_2 laser, 3 sessions every 3-4 weeks; optional extension to 6 sessions
Gold laser versus hyaluronic acid [5] Austria	Intravaginal Er:YAG laser, 2 applications (baseline and 1 month)
LARF/EPMLARF arm 2 [8,14,15] Brazil	Fractional CO_2 laser, 3 monthly sessions
Okui two-strategy laser cohort [16] Japan	Er:YAG vaginal laser plus Nd:YAG vulvar treatment, 3 sessions every 30 days
Fidecicchi hyperstacking pilot [17] Italy/Argentina	Er:YAG SMOOTH standard vaginal protocol, 3 sessions at 30-day intervals
VagLaser dose/histology programme [9,18] Denmark	Fractional microablative CO_2 laser, 5 monthly sessions
Quick/Ohio State cohort [19–21] United States	Fractional CO_2 laser, 3 treatments at 30-day intervals
Favier 24-month cohort [22] France	MonaLisa Touch fractional CO_2 laser, 3 sessions 4-6 weeks apart
Veron extended-follow-up cohort [23] France	Fractional microablative CO_2 laser (MonaLisa Touch), 3 monthly sessions
Salvatore five-session pilot [24,25] Italy	Microablative fractional CO_2 laser, 5 applications every 4 weeks
Siliquini BCS/healthy comparison [26] Italy	Fractional microablative CO_2 vaginal and vulvar laser, 3 treatments every 30 days
LAAVA pilot [27] Australia	Fractional CO_2 vaginal laser, 3 treatments 4 weeks apart
Arêas vaginal Er:YAG cohort [28] Brazil	Er:YAG 2940-nm vaginal laser, 3 sessions at 30-day intervals
Gambacciani/Levancini Er:YAG pilot [29,30] Italy	Nonablative Er:YAG SMOOTH vaginal laser, 3 applications every 30 days
Pieralli dyspareunia cohort [31] Italy	Fractional microablative CO_2 laser, 3 sessions

Study / family	Device-specific and protocol description as reported
Pagano 2016 cohort [32] Italy	Fractional microablative CO_2 laser, 3 cycles every 30-40 days
Pagano 2018 larger cohort [33] Italy	Fractional microablative CO_2 laser, 3 cycles every 30-40 days
Mothes dual-phase Er:YAG cohort [34] Germany	Single 10-minute dual-phase ablative/thermal Er:YAG treatment
Chung Korean cohort [35] Republic of Korea	Fractional CO_2 vaginal laser, 3 sessions 4 weeks apart
Ferreira Er:YAG cohort [36] Brazil	Er:YAG vaginal laser, 3 monthly sessions
Perrini dual-wavelength diode cohort [37] Italy	Combined 980/1470-nm diode vaginal laser, 3 sessions every 4 weeks
Lami CO_2 cohort [38] Italy	CO_2 laser, described by the study authors as non-ablative; 3 sessions
Lubián-López SSVL cohort [39] Spain	Non-ablative solid-state vaginal laser protocol
Hersant EPIONE feasibility cohort [40] France	Fractional CO_2 vaginal laser, 2 sessions
Gittens and Mullen endocrine subgroup [41] United States	Fractional CO_2 laser (MonaLisa Touch), 3 sessions 6 weeks apart
Becorpi clinical and biomarker cohort [42] Italy	Fractional CO_2 vaginal laser protocol
AFLT study for MMK [43] Japan	Ablative fractional CO_2 laser (SmartXide2), 3 sessions every 4 weeks

Supplementary Table S12. Adverse-event ascertainment and reported events by laser modality

Reported terms and severity categories are reproduced as closely as possible from the source reports; they were not harmonized across studies. Absence of a reported event does not establish safety when surveillance was not prespecified or fully described.

Study / modality	Safety population	Surveillance or ascertainment	Reported adverse events	Maximum reported severity / serious events	Principal limitation
LIGHT [6] — CO_2	84 randomized; 72 analyzed	Prospective sham-controlled trial; adverse events and serum estradiol assessed	Mild or moderate related events; no severe event; no estradiol increase in laser arm	No serious treatment-related event reported	Safety denominator and definitions were not uniform across reports
Jaber [7] — CO_2	34 comparative-phase completers	Events reported by randomized arm during the comparative phase	Spotting/discomfort: 5/19 laser vs 7/15 sham; one cystitis requiring antibiotics in each arm	No serious event reported	Small sample and short blinded follow-up
Gold [5] — Er:YAG	43 analyzed	Randomized active-comparator trial; author-reported adverse-effect assessment	No adverse effects reported	No serious event reported	Surveillance definitions were not detailed; 3-month follow-up
LARF/EPMLARF [8,14,15] — CO_2	70 randomized; 58 in main 10-month analysis	Prospective trial monitoring with clinical and histologic tissue assessment	Procedural pain and mild bleeding; some withdrawals because of pain; no burns or tissue damage	No serious treatment-related event reported	Event denominators varied across companion reports
Okui [16] — Er:YAG+Nd:YAG vs Er:YAG	204 propensity-matched	Retrospective follow-up with symptom and adverse-event reporting through 24 months	Transient heat, mild pain, discharge, edema, dryness, itching, and one transient de novo incontinence event	No persistent or serious event reported	Retrospective ascertainment and post-treatment selection
Fidecicchi [17] — Er:YAG	68 randomized	Only a detailed abstract was available to the review team	Safety outcomes not sufficiently detailed	Not classifiable	Definitions, denominators, and surveillance method unavailable
VagLaser [9,18] — CO_2	30 enrolled; 27 completed five sessions	Prospective tolerability assessment with linked post-treatment histology	Treatment described as well tolerated; no epithelial injury in histology report	No serious event reported	Clinical event details were limited in the abstract-only dose report
Quick/Ohio State [19-21] — CO_2	64 treated	Prospective follow-up with graded adverse-event reporting through 2 years	Grade 1-2 discharge and dryness	No grade ≥3 event reported	Marked attrition at extended follow-up
Favier [22] — CO_2	Breast-cancer subgroup n=26	Retrospective extended-follow-up assessment	One atypical endometrial hyperplasia in an HRT user at 3 years; causal relation not established	No serious treatment-related event reported	Selected responder sample and non-standardized ascertainment
Veron [23] — CO_2	46 included	Prospective cohort follow-up; adverse events reported during treatment and follow-up	No major adverse event reported	No serious treatment-related event reported	Incomplete extended follow-up and limited event definitions

Study / modality	Safety population	Surveillance or ascertainment	Reported adverse events	Maximum reported severity / serious events	Principal limitation
Salvatore [24,25] — CO_2	40	Prospective cohort monitoring during five sessions	No infection, worsening symptom, or other treatment-related adverse event recorded	No serious event reported	Small uncontrolled cohort
Siliquini [26] — CO_2	Breast-cancer subgroup n=45	Retrospective treated-cohort adverse-event reporting	No adverse event reported	No serious event reported	Surveillance method and event definitions not standardized
LAAVA [27] — CO_2	26	Prospective feasibility-study monitoring	No serious adverse event reported	No serious event reported	Small sample and 12-week follow-up
Arêas [28] — Er:YAG	24 analyzed	Prospective recording of complications after three sessions	One candidiasis and one acute cystitis; both resolved with treatment	No serious event reported	Short follow-up and small cohort
Gambacciani/Levancini [29,30] — Er:YAG	43 treated; 37 completers	Prospective cohort; general adverse-event reporting	No adverse event reported	No serious event reported	Surveillance procedures were not described in detail
Pieralli [31] — CO_2	50	Prospective treatment cohort with complication reporting	No major complication reported	No serious event reported	Safety assessment was not standardized
Pagano 2016 [32] — CO_2	26	Retrospective monitoring of procedural discomfort and adverse reactions	Transient procedural discomfort/reactions	No serious event reported	Short follow-up and retrospective ascertainment
Pagano 2018 [33] — CO_2	82 treated; 77 completed	Retrospective adverse-effect reporting across three cycles	Five participants did not complete all cycles; no serious event reported	No serious event reported	Reasons for non-completion and event grading incompletely detailed
Mothes [34] — Er:YAG	16	Retrospective complication assessment	No adverse complication reported	No serious event reported	Very small selected postoperative cohort
Chung [35] — CO_2	30 enrolled; 24 completed	Prospective cohort adverse-event assessment	No serious adverse event reported	No serious event reported	Twenty percent did not complete final assessment
Ferreira [36] — Er:YAG	12 enrolled; 8 at 1 year	Prospective clinical and tissue follow-up	No serious adverse event reported	No serious event reported	Very small sample and substantial attrition
Perrini [37] — dual-wavelength diode	Breast-cancer subgroup n=13	Prospective procedural-pain and adverse-event assessment	No significant adverse event reported; procedural pain assessed	No serious event reported	Small subgroup and short follow-up
Lami [38] — CO_2	26	Only a detailed abstract was available to the review team	No adverse event reported in the available abstract	No serious event reported	Full surveillance methods and denominators unavailable
Lubián-López [39] — other solid-state	30 eligible; 24 at 24 weeks	Prospective scheduled follow-up with side-effect reporting	No relevant side effect reported	No serious event reported	Loss to follow-up and limited event definitions
Hersant [40] — CO_2	20	Prospective feasibility-study monitoring of pain, bleeding, and other adverse effects	Mean procedural pain 4.7/10; two moderate bleeding events within 24 hours	No serious event reported	Small feasibility sample
Gittens/Mullen [41] — CO_2	Breast-cancer subgroup n=8	Retrospective subgroup report	No adverse event documented	Not adequately assessable	Very small subgroup; safety ascertainment not described
Becorpi [42] — CO_2	20	Prospective clinical cohort monitoring	No serious adverse effect reported	No serious event reported	Small cohort and limited follow-up for delayed harm
AFLT [43] — CO_2	7 reported in registry	Registry-results-only evidence	Insufficient event detail	Not classifiable	Methods, denominators, and extended follow-up results insufficient
